# Effects of *Brassicaceae* Isothiocyanates on Prostate Cancer

**DOI:** 10.3390/molecules21050626

**Published:** 2016-05-12

**Authors:** Silvia Novío, María Elena Cartea, Pilar Soengas, Manuel Freire-Garabal, María Jesús Núñez-Iglesias

**Affiliations:** 1 Lennart Levi Stress and Neuroimmunology Laboratory, School of Medicine and Dentistry, University of Santiago de Compostela, c/San Francisco, s/n, 15782 Santiago de Compostela, A Coruña, Spain; manuel.freire-garabal@usc.es (M.F.-G.); mjesus.nunez@usc.es (M.J.N.-I.); 2 Group of Genetics, Breeding and Biochemistry of Brassicas, Misión Biológica de Galicia (CSIC) Aptdo. 28, 36080 Pontevedra, Spain; ecartea@mbg.csic.es (M.E.C.); psoengas@mbg.csic.es (P.S.)

**Keywords:** angiogenesis, apoptosis, carcinogenesis, cell cycle, chemoprevention, epigenetics, isothiocyanates, metastasis, prostate cancer, therapy resistance

## Abstract

Despite the major progress made in the field of cancer biology, cancer is still one of the leading causes of mortality, and prostate cancer (PCa) is one of the most encountered malignancies among men. The effective management of this disease requires developing better anticancer agents with greater efficacy and fewer side effects. Nature is a large source for the development of chemotherapeutic agents, with more than 50% of current anticancer drugs being of natural origin. Isothiocyanates (ITCs) are degradation products from glucosinolates that are present in members of the family *Brassicaceae*. Although they are known for a variety of therapeutic effects, including antioxidant, immunostimulatory, anti-inflammatory, antiviral and antibacterial properties, nowadays, cell line and animal studies have additionally indicated the chemopreventive action without causing toxic side effects of ITCs. In this way, they can induce cell cycle arrest, activate apoptosis pathways, increase the sensitivity of resistant PCa to available chemodrugs, modulate epigenetic changes and downregulate activated signaling pathways, resulting in the inhibition of cell proliferation, progression and invasion-metastasis. The present review summarizes the chemopreventive role of ITCs with a particular emphasis on specific molecular targets and epigenetic alterations in *in vitro* and *in vivo* cancer animal models.

## 1. Introduction

Over the past several decades, research on the action of plant bioactive constituents has been focused predominantly on their benefits for human health. Today we can begin to explain why consuming a diet rich in vegetables and fruits may lead to a reduced incidence of certain diseases, such as cancer [1,2]. Plant secondary products have complementary and overlapping actions, including the modulation of detoxification enzymes and the stimulation of the immune system, the reduction of inflammation, the modulation of steroid metabolism, antibacterial and antiviral effects and antioxidant effects.

One group of vegetables that has drawn a great deal of attention are the vegetables from the family *Brassicaceae* (Cruciferae). The family *Brassicaceae* is a large group, having about 3000 species in 350 genera, including several types of edible plants, which are sometimes referred to as ‘the cabbage family’. The most well-known species within the *Brassicaceae* are edible vegetables such as *Brassica oleracea* (broccoli, cabbage, cauliflower, *etc.*); *B. rapa* (turnip); *B. napus* (canola, leaf rape); *B. nigra* (black mustard); *Sinapis alba* (white mustard); *Raphanus sativus* (radish); *Eruca sativa* (salad rocket) and *Diplotaxis tenuifolia* (perennial wall-rocket).

The benefits for human health associated to consumption of cruciferous plants could be explained, in part, by to their rich composition in secondary metabolites (*i.e.*, meaning that they are not essential for plant growth), frequently called phytochemicals. Glucosinolates are the main class of secondary metabolites that can be found in cruciferous crops. All glucosinolates have a common core structure that consists of a β-thioglucoside *N*-hydroxysulfate with a side chain R and a sulphur-linked β-d glucopyranoside moiety that derives from different types of amino acid precursors. Glucosinolates can be grouped into three chemical classes: aliphatic, indole and aromatic, according to whether their amino acid precursor is methionine, tryptophan or an aromatic amino acid (tyrosine or phenylalanine), respectively. There is a substantial amount of data compiled on the occurrence of glucosinolates in representative *Brassica* species [3,4,5]; to date, more than 120 individual glucosinolates have been isolated from species of the family *Brassicaceae* and the allied families [6]. Glucosinolate concentration varies according to the species and cultivar, tissue type, physiological age, plant health, environmental factors, insect attack and microorganism intrusion [3,4,7]. Upon cell damage, glucosinolates undergo hydrolysis by myrosinase to yield glucose, sulfate and aglucones that can undergo fragmentation and/or molecular rearrangement. Therefore, this process will yield isothiocyanates (ITCs), thiocyanates, oxazolindine-2-thione and nitriles, depending on the specific glucosinolate substrate, myrosinase isozyme, reaction pH and the presence of certain ions and the activity of specific protein factors, such as the epithiospecifier protein (ESP) [6]. Notably, nearly all of the protective activities of glucosinolates, among them that one against cancer, can be attributed to their hydrolytic products, of which the ITCs are prominent examples [7,8,9]. For this reason there is an increase in their value as therapeutic compounds to be used in medicine and their value as food supplements for human diet [10].

## 2. Biological Activity of Glucosinolates and ITCs: Their Role in Cancer Prevention

The first evidence of the benefits of glucosinolate degradation products for human health comes from investigations in the 1960s and 1970s, which used rodent models of chemical carcinogenesis [11]. Subsequently, this has been corroborated with models of genetic predisposition [12] and with xenografts [13]. Likewise, an extensive review of epidemiological studies published prior to 1996 reported that the majority (67%) of case-control studies found an inverse association between some type of cruciferous vegetable intake and cancer risk [14]. By way of example, Graham *et al.* [15] reported that the risk of cancer is increased among individuals with low consumption of cabbage, Brussels sprouts, and broccoli, and decreased among those with high consumption of these vegetables, and pointed out that these findings are consistent with the decreased number of tumors in animals challenged with carcinogens and fed compounds found in the same vegetables. Nowadays, it is known that this protection is not organ-specific and it has been seen in the lung, esophagus, stomach, colon, breast, bladder, pancreas, and prostate [16]. Likewise, this protective effect is attributed to subtoxic concentrations of glucosinolate degradation products. Kirsh *et al.* [17] have observed that 3–5 servings of broccoli or cauliflower per week could be cancer-preventive, however the therapeutical effective concentration has not been determined in clinical studies so far.

During the last two decades, ITCs have gained attention as they are responsible for the cancer chemopreventative properties attributed to cruciferous crops [18]. Thus, the anticarcinogenic effects of phenethyl isothiocyanate (PEITC) are being the object of study of several clinical trials. On the one hand, efforts are underway to determine its effectiveness in preventing lung cancer in smokers (clinicaltrials.gov Identifier: NCT00005883) as well as in depleting mutant p53 within the oral cells (clinicaltrials.gov Identifier: NCT01790204). On the other hand, Ravasco (clinicaltrials.gov Identifier: NCT02468882) suspects that this ITC could modulate breast cancer progression and recurrence.

Attempts to understand the mechanisms of action of ITCs began in parallel with studies demonstrating their protective effects in animal models of carcinogenesis. It is now widely recognized that these mechanisms are multiple, so carcinogenesis could be inhibited both in an early and a late state. These mechanisms include at least the following: alterations of carcinogen metabolism due to changes in the activities of drug-metabolizing enzymes; induction of cell cycle arrest and apoptosis; inhibition of angiogenesis and metastasis; changes in histone acetylation status; and oxidant activities [19].

The antioxidant and pro-oxidant activity of ITCs, both *in vivo* and *in vitro*, has been reviewed recently [19,20]. Although the oxidative damage induced by bioactive molecules derived from cruciferous vegetables is one of the most common and well-known cytotoxicity mechanisms by which they can kill cancer cells or at least retard the progression of this disease [19], the action of these compounds over the other mechanisms previously mentioned is gaining more interest. Thus, we will discuss the cancer chemopreventive role of glucosinolate degradation products in the following sections, with a particular emphasis on specific molecular targets and epigenetic alterations in *in vitro* and *in vivo* cancer animal models. In particular, we have mainly summarized the effects of PEITC, sulforaphane (SFN), benzyl isothiocyanate (BITC) and allyl isothiocyanate (AITC) in prostate cancer (PCa) among the different compounds derived from the hydrolysis of glucosinolates (Table 1).

## 3. Role of ITC in Cancer Epigenetics

The importance of epigenetics on cancer initiation and development is a growing area of interest. Different epigenetic changes, like aberrant DNA methylation, histone modifications, and microRNA profiles, can induce altered gene expression and functional changes, such as tumor suppressor genes silencing and/or activation of oncogenes [21], developing an important role in carcinogenesis [22,23,24,25,26,27]. DNA hypomethylation can facilitate genome instability and thus an enhanced expression of oncogenes, whereas DNA hypermethylation can silence tumor suppressor genes, transcription factors, and genes involved in the regulation of cell cycle and apoptosis [27]. DNA methyltransferases (DNAMTs) are involved in DNA methylation patterns [28] and are overexpressed in many cancers, such as PCa [29], lung cancer [30], leukemia [31], pancreatic cancer [32] or gastric cancer [33]. Histone molecules contribute to genome stability and gene transcription [34], and some transcriptional modifications (acetylation, deacetylation, methylation, phosphorylation and ubiquitination) can alter them [35] with consequences on cancer development [24,25,36,37]. Other epigenetic events have been described, the so-called “cancer progenitor cells” (CPCs), probably involved in the development of the metastatic properties of tumors [38]. These cells should develop from a population of normal human stem cells as a consequence of a multifactorial process including environmental, genetic and mainly epigenetic changes [38,39].

Since epigenetic deregulation appears at the startup and the development of cancer and it is potentially reversible, many authors have proposed epigenetic intervention strategies for cancer prevention and treatment [22]. Moreover, epigenetic biomarkers could be useful to diagnose cancer, as well as to establish a prognosis of the disease. The development of epigenetic drugs (“epi-drugs”) as well as the design of epigenetic diets against cancer may have a potential in the near future [40,41].

As previously described, cruciferous vegetables have demonstrated properties against cancer [42] that could be attributed to ITC compounds found in these plants, at least in part. ITCs may be regulators of DNAMTs, miRNAs, and inhibitors of histone deacetylases (HDACs) [42], affecting the uncontrolled cellular proliferation and the viability of various types of cancer cells like breast [43,44], leukemic [45], pancreatic [46], colon [47] or skin [48,49,50].

Among the cancers cited above, the concurrence of various aspects explains the interest in PCa and the need for new therapeutic strategies to treat it. Essentially, these aspects include that it represents one of the most common cancers in men, which is expected to increase with population aging, and that it can develop resistance to conventional therapy [androgen deprivation therapy (ADT) resistance] over the course of the disease that is associated with poor prognosis and metastasis [51]. Studies performed with PCa cells [27,51] provide evidence that ITCs may act as epigenetic modulators, thus having consequences on the initiation and progression of carcinogenesis [27,51,52]. Their effects can help restore cells to a more normal state [51].

SFN has been found to modulate some epigenetic mechanisms like DNAMT expression and DNA methylation in both normal and cancerous (androgen-dependent and androgen-independent) prostate cells [27,43,53,54]. These effects of SFN on DNA methylation can lead to the re-expression of some tumor suppressor genes that got silenced in cancer cells. Likewise, SFN can also inhibit HDAC activities upregulated in cancer [55,56,57,58]. In particular, SFN can inactivate the HDAC6, influencing the acetylation state of HSP90 (a key androgen receptor (AR) chaperone) attenuating AR signaling [58], and then androgen-dependent PCa cell growth.

The pathogenesis of PCa is dependent upon signaling through the activation by the androgen ligands testosterone or dihydrotestosterone of the steroid nuclear hormone AR, inducing or repressing gene expression through binding to chromatin at *cis* androgen response elements resulting in an increase of cell growth [59]. Since Huggins *et al.* evidenced in 1941 [60] the benefit of androgen deprivation in advanced metastatic PCa, androgen deprivation therapy (ADT) became a standard of care that continues to this day for patients with cancer recurrence after a definitive primary therapy, locally advanced disease or metastatic PCa. Nevertheless, although most patients initially respond favourably to hormonal therapy, the disease progresses to a more severe stage termed castration-resistant disease (CRPC) [59].

PEITC is another ITC with effects on PCa involving different epigenetic mechanisms. These include, like other HDAC inhibitors [61,62], the downregulation of AR expression at transcriptional and posttranslational levels [63]. This compound, as well as other ITCs and some metabolites, can decrease the levels and activities of cdk/cyclins and increase the activity of the transcriptional factor Sp1 [64,65], which is a regulator of AR expression in PCa [51,66]. PEITC can also restore the expression of the detoxifying phase II enzyme π-class glutathione-S-transferase (GSTP1), which is silenced in the vast majority of prostate tumor cells [51,67,68], via CpG island demethylation [69]. Studies using TRAMP mice also showed that PEITC treatment can inhibit the CGI hypermethylation that occurs at the early stage of prostate carcinogenesis [51].

## 4. *In Vitro* Studies

At the molecular level, the PCa cells can acquire mutations or epigenetic modifications that trigger the malignant phenotype with the capacity of uncontrolled growth, survival, and invasion-metastasis. As a result, the activation of receptors and growth factors, signaling proteins, kinases, transcription factors and coregulators, and multiple proteases required for tumor progression can occur. Conceivably, PCa cells could be interrupted along these different key points, which have been established as a potential target for PCa therapy [70].

This section, focusing on *in vitro* studies, highlights the possible molecular mechanisms of action of ITCs against specific targets in three PCa cells (DU 145, PC3, and LNCaP cells). This triad of cells represents the gold standard of PCa cell lines in culture [71]. These cells differ fundamentally in terms of p53 and Bax status [72,73] and androgen sensitivity [72], where DU 145 is p53 mutant and Bax negative but PC3 and LNCaP are p53 null and p53 wild-type, respectively. Furthermore, LNCaP are androgen-dependent cells and DU 145 and PC3 are androgen-independent cells, being PC3 more aggressive cancer cells.

Some evidence suggests that ITCs not only suppress PCa development during the “post-initiation” phase of cancer via the induction of cell cycle arrest and apoptosis (see Section 4.1 and Section 4.2 below) but also the invasion-metastasis (see Section 4.3) and ADT resistance (see Section 4.4) in a dynamic and cell type-specific manner. However, the effect of the ITCs should not be extrapolated between them.

### 4.1. Effects on Cell Cycle Proteins: Cell Cycle Arrest

**Figure 1:**
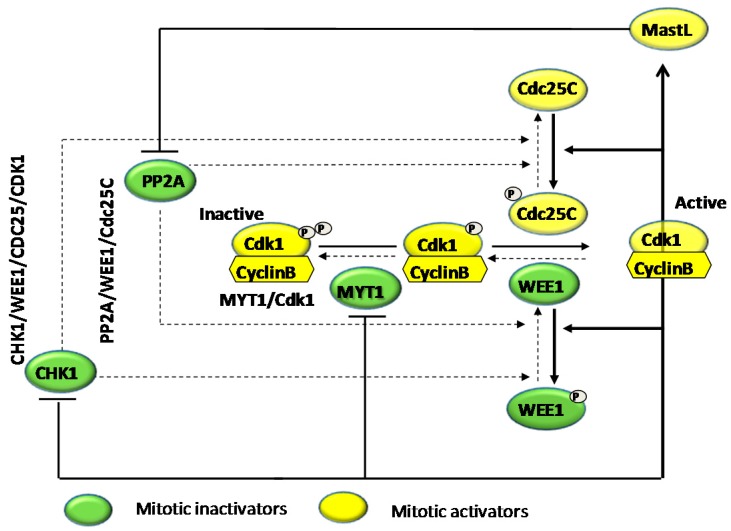
Transition from G2-checkpoint arrest to mitosis. For simplicity, a solid line represents how CDK1–cyclin B triggers entry into mitosis and a dashed line represents Cdk1-inactivating pathways (CHK1/WEE1/Cdc25C/Cdk1, MYT1/Cdk1, and PP2A/WEE1/Cdc25C). Abbreviations: Cdk1, cyclin-dependent kinase 1; CHK1, checkpoint kinase 1; MYT1, membrane associated tyrosine/threonine 1 kinases; PP2A, protein phosphatase 2.

Mitosis regulators can push cells forward into mitosis or hold them in G_2_-arrest. Both WEE1 and its complementary counterpart, cell division cycle 25C (Cdc25C), represent the main switch for mitosis by means of double-activating feedback loops. Thus, in order to facilitate the progression of mitosis, activated cyclin-dependent kinase 1 (Cdk1) activates and inactivates its activators (Cdc25C and MastL) and inactivators (WEE1 and MYT1 (membrane associated tyrosine/threonine 1) kinases), respectively [74,75,76]. There are three parallel Cdk1-inactivating pathways: CHK1/WEE1/Cdc25C/ Cdk1, MYT1/Cdk1, and PP2A/WEE1/Cdc25C [74] (Figure 1).

The Cdc25 family (Cdc25A, Cdc25B and Cdc25C) is upregulated in PCa and its expression level is positively associated with the Gleason score and disease metastasis [77,78,79]. Moreover, it is an AR coregulator suppressing the AR transcriptional activity [77,79]. In contrast, WEE1 can be considered a tumor suppressor, which, being lost in normal prostate epithelial cells, increases the susceptibility to genetic aberrations and carcinogenic transformation [76].

Besides WEE1, there are other tumor suppressors such as p21 or p53 that fail to restrict cell cycling in PCa. The p21 protein (known also as WAF1, CAP20, CIP1, and SDI1), a member of the CIP/KIP family of Cdk inhibitors, is a tumor suppressor as well as a cell cycle inhibitor protein [80]. Additionally, the expression of p21 appears associated with the progression of androgen-independent prostate cancer (AIPC) [81]. The p53 is a downstream target of Chk2 kinase governing the G_2_/M transition by transcriptional regulation of Cdk inhibitor p21. Additionally, the p53 status of PCa cells may determine the response to radiation therapy; including the number and the proportion of genes upregulated or downregulated after irradiation [72,80]. Furthermore, p53 is considered a tumor suppressor that is a key target in cancer therapy [82].

AITC (20 µM) induces G_2_/M phase arrest both in androgen-dependent (LNCaP) and -independent (PC3) PCa cells, but not in the normal prostate epithelial cell line (PrEC). This effect, in both PCa cell lines, is attributed to a decreased expression of the proteins involved in G_2_/M progression: Cdk1, Cdc25B and Cdc25C, participating also cyclin B1 protein in LNCaP cells [83].

There are different mechanisms of action for PEITC that could contribute to G_2_/M phase arrest in PCa cells [66,84,85,86,87]: (i) downregulation of Cdk1 and cyclin B1 protein expression in LNCaP cells. This effect is extensible to the PEITC metabolite, PEITC-NAC (*N*-acetylcysteine conjugate of phenethyl isothiocyanate), which is produced as a result of absorption and metabolism in humans by conjugation of glutathione followed by conversion via the mercapturic acid pathway to a NAC conjugate [85]; (ii) upregulation of p53 and WEE1 expression and downregulation of Cdc25C protein in DU 145 cells [86]; (iii) downregulation of c-Myc in PC3, relieving the suppressive effect on the promoter p21 [66]; (iv) proteasome-mediated degradation of Cdk1 and Cdc25C correlated with the increase of the accumulation of Tyr^15^ phosphorylated (inactive) Cdk1 in PC3 cells [84].

It seems that SFN arrests cell cycle because it induces: (i) a decrease in protein levels of cyclin D1, cyclin E, Cdk4, and Cdk6 in LNCaP cells [88]; (ii) checkpoint kinase 2-mediated phosphorylation of Cdc25C, inducing its sequestration in the cytosol in DU 145 cells [89]; (iii) c-Jun N-terminal kinase activation in DU 145 cells [89,90]; (iv) induction of p21 in PC3 and LNCaP cells regardless of p53 dependent and independent contexts [75]. SFN (20 µM) induces p53 [55] and p21 in LNCaP cancer cells [55,88], but the induction of p21 seems to be independent of p53, since it occurs in the absence of the induction of p53 and Ser15 phosphorylation, and it is probably associated with the inhibition of HDAC activity. In accordance with this aspect, SFN induces cell cycle arrest which is not substantially modified by the knockdown of p53. Also, SFN induces S phase arrest in LNCaP cells. It seems due to induction of cyclin B1 and down-regulation of Cdk1 and Cdc25C [88]. BITC induces G_2_/M phase arrest in DU 154 cells by increasing WEE1 levels and decreasing cyclin B1 and Cdc25C proteins levels [91].

**Figure 2:**
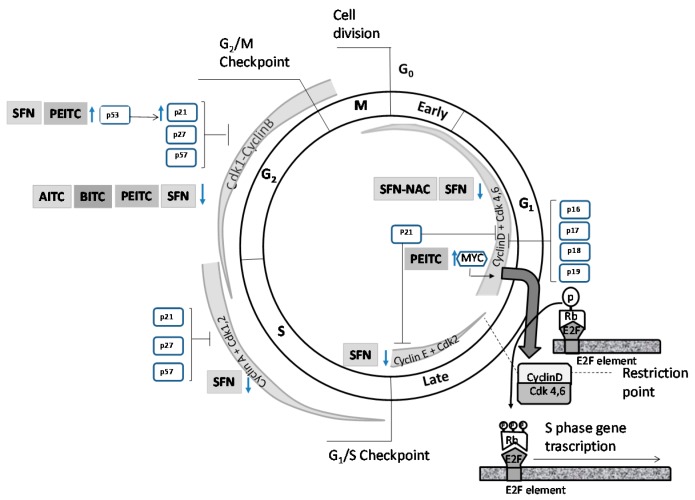
Effects of ITCs *in vitro* on cell cycle regulation by Cdk/cyclin holoenzymes and CKIs. Abbreviations: ↑, enhanced expression or protein levels; ↓, reduced expression or protein levels; AITC, allyl isothiocyanate; BITC, benzyl isothiocyanate; PEITC, phenethyl isothiocyanate; SFN, sulforaphane; SFN-NAC, *N*-acetylcysteine conjugate of sulforaphane.

The effects of ITCs on mitosis are not extrapolatable between them. Subtle differences in the chemical structure may be responsible for the differences in their effects [92]. It is suggested that the effect of ITCs on cell cycle is structure-dependent. Thus, PITC, a structural analogue of PEITC, losing the –CH(2) spacers that link the aromatic ring to the –N=C=S group, has no effect on cell cycle arrest or apoptosis, while PEITC has an effect on these parameters in PC3 [84] or DU 145 cells [93]. Likewise, the characteristics of the PCa cells could also influence the effect of a particular ITC. It seems that, besides an overall effect of SFN on the expression of cell cycle related genes, there is a specific effect depending on the prostatic cell line and perhaps the state of cancer progression [94]. Table 2 and Figure 2 summarize the possible effect of ITCs on mitosis regulators, their substrates or both, according to the type of ITCs and prostate cell line.

### 4.2. Induction of Apoptosis

#### 4.2.1. Intrinsic and Extrinsic Pathway

Type I programmed cell death (PCD) or apoptosis is carried out by activating both extrinsic and intrinsic pathways [97]. It seems that certain ITCs could induce apoptosis in PCa cells by acting on both pathways (Table 3).

The intrinsic pathway is triggered by different stimuli such as stress, resulting in the activation of Bax [B-cell lymphoma-2-like protein 4 (Bcl-2-like protein 4)] (via the activation of Bcl-2 BH3-only protein), the production of reactive oxygen species (ROS) and the ceramide that serve as second messengers acting on the mitochondria. This causes the release of mitochondrial apoptogenic factors cytochrome c, endonuclease G (Endo G) and apoptosis iducing fctor (AIF). Cytochrome c is combined with pro-caspase 9, dATP and APAF-1 to form the apoptosome that triggers the apoptosis through the activation of caspase 9 which then activates the executioner caspase 3 [97].

PEITC is involved in ROS production [86,102] derived from the inhibition of oxidative phosphorylation (OXPHOS), thus causing the activation of proapoptotic protein Bax (LNCaP and PC3 cells) [102] and the inhibition of complex III activity. The latter could be due to the fact that being a hydrophilic molecule could modify covalently sulfhydryl groups of complex III of the mitochondrial respiratory chain [103]. In addition, PEITC increases the mitochondrial release of cytochrome c and Endo G [86]. Similarly, SFN-induced apoptosis is initiated with the genesis of ROS and regulated by Bax and Bak [90,104]. ROS production is followed by the disruption of mitochondrial membrane potential and cytosolic release of cytochrome c in PC3 cells [104].

The extrinsic pathway is mediated by the activation of cell-death receptors (TNFR superfamily) that involves the recruitment and activation of caspases 8 and 10 (initiator caspases), which, in turn, induces the activation of caspase 3 (effector caspase) through the formation and activation of the death inducing signaling complex (DISC). The cleavage of death substrates by caspase 3 is the main executor of apoptosis hallmark (DNA fragmentation, nuclear fragmentation, membrane blebbing and other biochemical and morphological changes) [97]. Suppression of caspase 3 expression in PCa cells markedly decreases their sensitivity to apoptosis, thus contributing to cancer progression [105].

It has been shown that PETIC would act not only via the mitochondria in order to reduce mitochondrial membrane potential (ΔΨ_m_) and to increase Ca^2+^, but also through the extrinsic pathway by increasing the activity of caspases 3, 8 and 9 in DU 145 cells [86]. However, this ITC actives caspase-8 and -9 pathways (10 µM) in PC3 cells [84].

Treatment with BITC (DU 145 cells) promotes apoptosis via the mitochondrial signaling pathway triggered by ROS production (12 h post-treatment) followed by Ca^2+^ increase, ΔΨ_m_ decrease and AIF and Endo G release. Additionally, BITC increases caspase 3, 8 and 9 activity [91].

There is growing evidence that the extrapolation of the anti-apoptotic mechanism of action between ITCs is not admissible [92]. PEITC, but not PITC, induces apoptosis by both caspase-8- and -9-mediated pathway, which is decreased by specific inhibitors of caspase-8 and caspase-9 and general caspase inhibitor. It is considered that the expression of Bcl-2 fails to confer resistance to apoptosis induced by PEITC [84]. However, SFN activates caspases to trigger apoptosis both in DU 145 [64] and PC3 human androgen-independent cells [99].

#### 4.2.2. Anti-Apoptotic/pro-Apoptotic Proteins

It is well known that the Bcl-2 protein family is an important gatekeeper of the apoptotic response. This family includes anti-apoptotic proteins (such as Bcl-2, Bcl-X_L_ or BLL-B) or pro-apoptotic proteins (Bax, Bak, *etc.*) interacting among them. The former interact with and inhibit pro-apoptotic proteins; the latter cause the release of cytochrome c from the mitochondria by inducing the activation of caspases to execute cell death program [97].

Blc-2 protein expression varies depending on the progression stages of PCa cells [106]. Bcl-2 is not expressed in normal epithelial cells but its overexpression in LNCaP cells protects them from apoptosis and confers resistance to androgen ablation treatment [107]. Moreover, the upregulation of Blc-2 is required for the progression of LNCaP cells from the androgen-dependent to the androgen-independent state [108] and predicts recurrence and poor survival of localized cancer after radical prostatectomy [109,110]. AITC treatment (20 µM) of PC3 and LNCaP cells results in a significant reduction in the levels of Bcl-2 in both cell lines but only reduces the expression of Bcl-X_L_ in the LNCaP cells [83]. To equal treatment regimen (10 µM), PEITC and PITC induce apoptosis or not in PC3 cells, respectively. The apoptosis induced by PEITC is attributed to the reduction that it produces in Bcl-2 (more than 50% at 24 h) and Bcl-X_L_ (more than 40% at 24 h). However, the sensitivity of these cells to PEITC-induced apoptosis is not influenced by the overexpression of Bcl-2 [84]. These findings, if taken together, support the hypothesis that the effect of ITCs is structure-dependent. BITC treated PC3 cells show a decreased expression of Bcl-2 from 6 to 48 h post-treatment [98]. The Bcl-2/Bax ratio (intracellular suppressor of apoptosis/apoptotic agonist) in cells determines the existence or absence of apoptosis [111]. PITC induces the expression of Bax, but inhibits the expression of Bcl-2 (DU 145 cells), thus contributing, at least in part, to the activation of caspase 3 and to the activation of the intrinsic apoptosis pathway [86]. SFN upregulates Bax [99] in PC3 cells and downregulates the expression of Bcl-2 in DU 145 cells [64,99]. SFN causes Bax activation in LNCaP (10 µM, 20 µM, 40 µM) and PC3 (40 µM), showing LNCaP more sensitivity to SNF-induced apoptosis. The differential sensitivity towards SFN-induced apoptosis could be attributed to the difference in Bax activation profile or androgen responsiveness among these cell lines. P53 knockdown in LNCaP cells does not confer protection against SFN-induced apoptosis. Therefore, it seems that the difference in p53 status among these cells does not contribute to the difference in sensitivity to death [100].

#### 4.2.3. Inhibitor of Apoptosis Proteins (IAPs)

Regardless of the members of the family Bcl-2, other proteins can antagonize apoptosis, including IAPs, FLIPs and Faim3 [97]. IAPs include neuronal apoptosis inhibitory protein (NAIP), IAP-like protein 2 (ILP2), cellular IAP 1 (cIAP1), cIAP2, baculoviral IAP repeat containing ubiquitin-conjugating enzyme (BRUCE), X-linked inhibitor of apoptosis protein (XIAP), survivin and livin (ML-IAP), among others [112]. IAPs play an essential role in the inhibition of apoptosis since they cause: direct inhibition of caspases (XIAP) [97,112], kidnapping of pro-apoptotic molecules such as SMAC/DIABLO (cIAP1/2, survivin, livin), activation of the pro-survival NF-κB pathway (cIAP1/2, XIAP), ubiquitin-mediated degradation and non-degradative inactivation of caspases (cIAP1/2, XIAP), *etc.* [112].

The evasion of apoptosis in the PCa occurs both by an alteration in function and the levels of apoptosis regulators. XIAP and survivin are overexpressed in human PCa [113,114]. This fact is associated with PCa recurrence [114]. Also, the expression of cIAP12 correlates positively with the pathological T stage and the positive surgical margins. Likewise, there is correlation between survivin expression and perineural invasion [105]. PEITC exposure causes apoptosis associated to XIAP and survivin protein downregulation, regardless of the p53 status or the sensitivity to androgens (observed both in PC3 and LNCaP cells), being more sensitive to PEITC-induced apoptosis LNCaP cells in comparison to PC3 ones (2.5–5 µM). Conversely, overexpression of survivin protects these cells from the pro-apoptotic effect of PEITC [92] and SFN [101].

### 4.3. Inhibition of Migration and Metastasis

**Figure 3:**
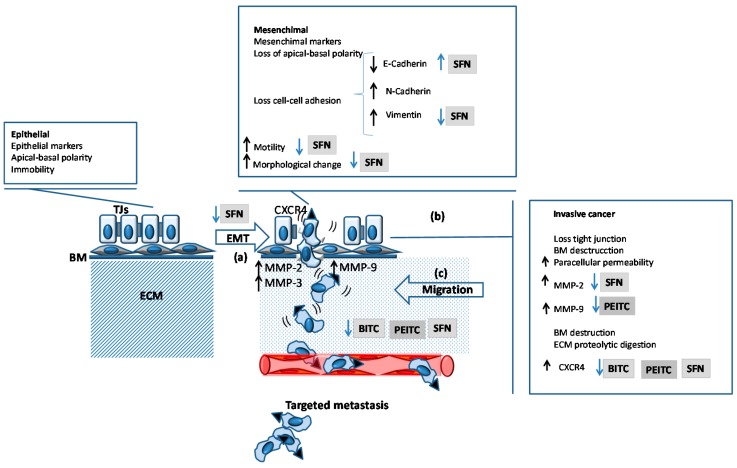
Schematic representation of the inhibitory effects of ITCs on EMT and invasion-metastatic mechanism in PCa cells *in vitro*. (**a**) During EMT epithelial cells decrease adhesion, change their morphology, polarity and position. EMT is characterized by a dowregulation (↓ bold arrow) and upregulation (↑ bold arrow) of genes that are characteristic of an epithelial and mesenchymal phenotype, respectively; The invasive (**b**) and migration (**c**) capacities are increased in the cells overexpressing CXCR4, MMP-2, MMP-9, MMP-3 (↑ bold arrow). Inhibitory effects of ITCs are represented by blue arrows (↑, enhanced expression, activity or protein levels; ↓, reduced expression, activity or protein levels). Abbreviations: BITC, benzyl isothiocyanate; BM, basement membrane; CXCR4, chemokine receptor type 4; ECM, extracellular matrix; EMT, epithelial- mesenchymal transition; MMP-2, matrix metalloproteinases (gelatinase-A); MMP-9, matrix metalloproteinases (gelatinase-B); PEITC, phenethyl isothiocyanate; SFN, sulforaphane; SFN-NAC, *N*-acetylcysteine conjugate of sulforaphane; TJs, tight junctions.

Epithelial mesenchymal transition (EMT) is a key process in carcinogenesis and the metastatic PCa [115], involving loss cell polarity and cell-cell adhesion, acquisition of cell mobility, downregulation of E-cadherin, upregulation of vimentin, *etc.* (Figure 3a). As a consequence, PCa cells can invade (Figure 3b), migrate and metastasize (Figure 3c) [116,117].

The invasiveness of organ-confined Pcas is modulated by androgens, acting on programs of gene expression by transcription factor-encoding genes of the ETS family (ERG, ETV1, *etc.*) [118]. When organ-confined tumor cells overexpress ERG and present transcriptional upregulation of their downstream target chemokine receptor type 4 gene (CXCR4), increased cell motility is observed [119]. Both CXCR4 expression [120,121] and cell motility are modulated by androgens [121]. In particular, LNCaP cell motility is modulated by androgens in a dose-dependent manner [121]. PEITC, BITC and SFN suppress CXCR4 expression and cell migration in LNCaP, 22Rv1 (human prostate carcinoma epithelial cell line), C4-2 (same phenotype as LNCaP), and PC3 (Figure 3c) [122].

It seems that SFN reduces the levels of proteins required for EMT as well as differentiation, self-renewal, tumorigenesis, migration and metastasis in DU 145 and PC3 cells [123]. In particular, SFN (15 µM) inhibits migration and invasion by changing the morphology of DU 145 cells and by activating ERK1/2 and downstream signaling. These events lead to upregulation of E-cadherin and downregulation of CD44v6, which reduce MMP-2 expression and activity [124].

Likewise, it is thought that the SFN may act on the expression of MMP-3 mediated by Notch signalling. This is a complex involving interplay among receptors (Notch1, Notch2, Notch3, and Notch4) and ligands (Jagged1, Jagged2, Delta-like and ligands [Dll1, Dll3, and Dll4]), which play a crucial role in PCa development and metastasis [125,126]. Notch overexpression has been associated to PCa metastasis and EMT [127]. Conversely, the knockdown of Notch1 inhibits the invasion of PCa cells associated to the inhibition of MMP-9 [127]. SFN (10 or 20 µM) inhibits cell migration and activates Notch signaling, which is characterized by Notch1, Notch2, and Notch4 cleavage (active form) and increased transcriptional activity in PC3, LNCaP and LNCaP-C4-2B, regardless of the androgenic response or p53 status. Nevertheless, knockdown of Notch1, Notch2 or Notch4 has not a relevant effect on SFN-mediated inhibition of cell migration [128]. In line with these data, the transition from LNCaP to LNCaP−C4-2 cells (androgen-independence) does not affect PEITC-mediated changes in Notch signaling components in a relevant way [129]. The knockdown of Notch2 confers protection against PEITC-induced apoptosis in LNCaP or PC3 cells. Also, the knockdown of Notch2 increases PEITC-mediated inhibition of LNCaP and PC3 cell migration, attributed to downregulation in the expression of MMP-9 and urokinase plasminogen activator [129].

### 4.4. ADT Resistance

Although androgen deprivation constitutes the standard therapy in PCa, tumor cells may develop resistance to it. Certain ITCs may have some inhibitory effects on some of the main mechanisms responsible for this resistance, as shown below.

It is postulated that, because of their capacity for self-renewal and differentiation and apoptosis-resistance, CSCs in PCa could be responsible not only for the cancer formation, but also for the progression and metastasis. What is more, they could be resistant to chemotherapy and be responsible for the recurrence after treatment [123,130,131] and resistance to ADT [132]. The SFN seems to decrease the ability of self-renewal and spheroidal growth around 50% and 80%, respectively [123].

In a normal prostate gland, androgen-dependent AR signaling has a counterbalanced effect on epithelial cells growth; acting on the stromal cells stimulates the release of andromedins inducing andromedin-driven epithelial cell growth, whereas it causes G_0_ growth arrest within epithelial cells. However, the suppressor role of growth in normal cells is transformed into oncogenic in PCa cells mediated by c-Myc [133]. PEITC can reduce the growth of LNCaP cells mediated by the expression of c-Myc [66].

Furthermore, androgen sensitivity of PCa cells could be, at least in part, conditioned by SFN-induced changes on a broad spectrum of genes expression. Specificity protein 1 (Sp1) transcription factors are overexpressed in PCa and are associated with poor prognosis [134]. PCa cells overexpress *in vitro*, among others, three categories of genes under Sp1 upstream or downstream control, which are involved in the regulation of apoptosis, cellular response to stress, and cell cycle [94]. Sp1 could be a major transcriptional mediator of SFN-induced changes in these genes, depending on the cell line [normal (PrEc), early (LNCaP) or on late-stage PCa (PC3)] and the time of exposition (6 h and 24 h). In LNCaP cells, a broad spectrum of genes upstream (p53, NFκB, c-Myc, E2F1, BRCA1, *etc.*) and downstream (EGFR, p21, cyclin D1, cyclin E2, Cdk4, Cdc25A, E2F1 and p21) of Sp1 are altered. Only in PC3 cells, SFN treatment causes a strong increase in the expression of pro-apoptotic (Bid, Smac/Diablo, and ICAD), cell migration, extracellular matrix, and angiogenesis-related genes. In LNCaP cells, the predominant effect is related to the cell cycle arrest [94].

Signal transducers and activators of transcription (STATs) together with androgens, RAS/MAPK, PI3K/AKT and Notch signaling pathways contribute to modulate PCa CSCs chemoresistance and self-renewal [135]. Specifically, STAT activity, with the induction of target genes, promotes proliferation at the time that inhibits the apoptosis of tumor cells, increases angiogenesis and facilitates tumor immune evasion [136]. STAT3 integrates different signals involved in the metastatic castration-resistant PCa (mCRPC), such as the reactivation of AR or EMT and also mediates the interaction of tumor cells with the microenvironment and immune activation, thus allowing PCa cells to escape immune detection and to promote an enabling environment for immune growth and tumor metastasis [137]. Constitutive STAT3 is required for the survival of prostatic tumor cells and its inhibition reduces cell growth and promotes apoptosis [138]. Direct or indirect targeting the STAT3 signaling pathway has been explored in order to counteract cancer [139]. It is thought that hyperactive STAT3 is oncogenic in PCa [140] and has been observed in around 50% of PCa [141]. Moreover, high STAT3 activation is associated with increased PCa malignancy with high Gleason score [141].

STAT3 activation in PCa cells is mediated by the activation of different upstream Janus kinase (JAK) depending on the cell line, being JAK2 and JAK1 for DU 145 and NPR-145 cells, respectively. However, the direct inhibition of STAT3 induces apoptosis in both cell lines [138]. IL-6 can act as a growth factor autocrine and paracrine in CaP cells that activates JAK/STAT3 and mitogen-activated protein kinase (MAPK) pathways. Androgen-resistant cells (DU 145 and PC3) constitutively express IL-6 [141]. On the other hand, activated STAT3 phosphorylation and the JAK/STAT3 pathway ensure the AR signal maintenance and increase IL-6-induced AR activation [142]. STAT3 activation by IL-6 may be abolished by CK2 inhibition [143].

PEITC reduces constitutive and IL-6-induced JAK2/STAT3 phosphorylation in DU 145 cells [87]. It seems that PEITC, but not PITC, suppresses the proliferative activity by the inhibition of JAK/STAT3 activation in DU 145 cells [93]. However the mechanism has not been fully elucidated. A synthetic analogue of broccoli-derived L-isomer of SFN (D, L-SFN) not only decreases the constitutive (DU 145 cells) and IL-6-induced STAT3 phosphorylation (DU 145 and LNCaP cells) but also its upstream regulator JAK 2. While the effect on the IL-6-induced STAT3 phosphorylation can be seen both in DU 145 as LNCaP cells, the effect on constitutive phosphorylation only occurs in DU 145 cells [144]. On the other hand, the phosphoinositide 3-kinase (PI3K)-AKT-mammalian target of rapamycin or the mechanistic target of rapamycin (mTOR) signaling pathway is involved in the ADT [145]. SFN treatment decreases mTOR signalling in PC3 cells [101] and likewise, BITC inhibits mTOR activity in androgen-independent PCa cells in a dose-dependent manner [98].

## 5. *In Vivo* Studies

Although cellular studies constitute an important step in the development of drugs, demonstrating preventative [146,147,148] and/or therapeutic [146,147] efficacy in suitable animal models and validating cellular observations *in vivo* are a condition *sine qua non* for clinical trials investigating potential anticancer agents. Inconsistencies in results between *in vitro* and *in vivo* systems concerning the effect of ITCs have been observed (Table 4) and although the reasons for these discrepancies are unclear, the following explanations have been suggested:

(i) the dose of ITCs: the necessary concentration of ITC might not have been administered *in vivo*, thus being required a more intensive dosing regimen to elicit a response;
(ii) the metabolism of ITCs: the exposure of cultured tumor cells to ITCs can lead to a very high intracellular accumulation of them, which may not be possible *in vivo* due to the rapid excretion of the conjugates of ITCs;
(iii) the activation of *in vivo* (but not *in vitro*) mechanisms to counteract the anticancer effect of the compounds (p.e. induction of prosurvival pathways, increased expression of IAPs, *etc.*).

These observations raise caution with regard to the extrapolation of results between *in vitro* and *in vivo* assays.

The selection of appropriate biological models, with optimal sensitivity and specificity, is an important aspect in any research setting. Although an ideal animal model for PCa does not exist, the transgenic adenocarcinoma of the mouse prostate (TRAMP) model closely mirrors the pathogenesis of human PCa. One of the major strengths of this model is that the cancer arises from normal prostate epithelial cells in their natural tissue microenvironment and furthermore, it has a well-defined course of disease progression with resemblance to human PCa development, this is metastasis to distant sites, progression to androgen independence, and neuroendocrine differentiation [12].

Knowing the pharmacokinetics of ITCs is critical. In relation to this aspect, ITCs-mediated anticancer effects in animal models occur at micromolar concentrations, which are achievable in humans through a dietary intake of cruciferous vegetables. For instance, it has been estimated that consumption of one ounce of watercress could yield up to 60 µmol of PEITC [153]. Furthermore, dietary ITCs administration appears to be safe and well tolerated. Xiao *et al.* [150] have shown that the PEITC-supplemented diet (12 µmol PEITC/day) does not result in any toxic effects as evidenced by no change in the body weight or the presence of signs such as impaired movement and posture, indigestion or diarrhea and areas of redness or swelling.

There are more than one hundred naturally occurring ITCs, and of these, PEITC and SFN, alone or in combination with other compounds (docetaxel [154], curcumin [146,155], tumor necrosis factor (TNF)-related apoptosis ligand (TRAIL) [123,147]), have received the most attention. When combined with other chemopreventive agents, ITCs can improve the efficacy of conventional therapies and reduce their effective dose, thereby ameliorating untoward side effects. For example, Shankar *et al.* [147] have observed that the combination of SFN and TRAIL is more effective in inhibiting tumor growth, invasion, metastasis and angiogenesis and inducing apoptosis than TRAIL alone. In relation to the apoptotic effect, it has been observed that the treatment of mice with TRAIL results in an enhanced expression of proapoptotic proteins (Bax, Bak), higher caspase-3 and caspase-8 activities, and an inhibited expression of antiapoptotic proteins (Bcl-2 and Bcl-X_L_); these effects are higher when SFN is coadministered with TRAIL.

The mechanisms behind the anticancer effects of ITCs are not fully understood, but known responses to these natural products in *in vivo* assays include: (i) prevention of cancer development in animal models [12,65,99,146,147,148,149,150,151,152,154,155,156,157,158,159,160,161]; (ii) suppression of cancer cell viability in association with apoptosis induction [12,148,152,155] and Atg5-dependent autophagic cell death [12,157]; (iii) inhibition of metastasis [12,147,151,152]; (iv) inhibition of angiogenesis [12,147,148].

### 5.1. Prevention of Cancer Development in Animal Models

ITCs have proved to be effective chemoprotective agents in PCa in subcutaneous and orthotopic xenograft models of cancer in nude mice and *in ovo* CAM-assays, as well as transgenic animal cancer models, and their effect has been demonstrated both by oral administration [12,65,99,147,148,149,150,151,152,154,155,156,157,158,159,160,161] and i.p. injection [146,162]. Oral gavage of 5.6 µmol SFN is highly effective in suppressing the growth of PC3 xenografts in male nude mice thrice weekly; 10 and 20 days after starting therapy, the average tumor volumen in SFN-treated mice is >50% and ~71%, respectively, lower than that of control mice [99]. Additional studies using TRAMP mice showed that a similar dosing regimen (6 µmol SFN, three times/week) inhibits PCa progression, this is, it prevents the incidence (23%–28%) and/or burden (24%–44%) of prostatic intraepithelial neoplasia and well-differentiated carcinoma in the dorsolateral prostate of SFN-treated mice compared with that of control mice [152].

### 5.2. Suppression of Cancer Cell Viability in Association with Apoptosis and/or Autophagy Induction

ITCs-mediated inhibition of prostate carcinogenesis is associated with a reduced cellular proliferation [12,65,123,147,152,155,160,161,162] and the induction of apoptotic [12,65,92,99,146,148,150,152,154,155,156,159] and autophagic [12,157] cell death.

#### 5.2.1. Cellular Proliferation

ITC administration decreases the proliferation of AIPC cells, although it is ineffective in androgen-dependent ones [148] (Table 5). Oral gavage of SFN causes a statistically significant decrease in cell proliferation in the DLP of TRAMP mice, as evidenced by the reduced expression of the molecular proliferation marker PCNA (~40 % lower in mice treated with SFN compared with control mice) [152], which serves as a requisite protein for DNA polymerase δ-driven DNA synthesis and is cell cycle regulated [163]. Likewise, other proliferation markers, such as Ki-67, are affected by ITC treatment [12,123,147,161].

ITCs impede the progression of PCa at least by downregulating the Akt signaling pathway, which is involved in amplifying cell-survival signals by inactivating its downstream targets, such as members of the forkhead family [164]. Barve *et al.* [155] have shown reduced expression levels of PDK1, Akt and FKHR proteins in the prostatic tissues of TRAMP mice that were fed a diet supplemented with PEITC compared with mice that received control diet. Furthermore, the induction of insulin-like growth factor binding proteins (IGFBP) through regulation of inflammatory pathways could result in lowered tumor growth. In this respect, ITCs may decrease inflammation through modulation of IL-6 mediated pathways [147] that involves control of IGFBP3, thus resulting in the regulation of tumor cell proliferation [160].

On the other hand, growth inhibition of tumors is also associated with a reduction in cells undergoing mitosis due to the accumulation of the inactive Cdk1/cyclin B kinase complex. Thus, Srivastava *et al.* [162] have observed accumulation of cells in G_2_/M phase in the tumors of AITC-treated mice when compared with control tumors because of the lack of activation of Cdk1 mediated by Cdc25B and Cdc25C. However, it is also possible that cell cycle arrest occurs at another moment of it, such as G_1_ to S phase transition [65,147].

#### 5.2.2. Apoptosis

Apoptosis induction is regarded as an important mechanism for ITC-mediated inhibition of the development of AIPC [65,99]; however, like it was commented previously in relation to cellular proliferation, these natural compounds lack effects on androgen-mediated pathways [148]. Thus, the percentage of apoptotic cells is significantly higher (~3.3-fold) in PC3 tumor sections from SFN-treated mice than in controls [99]; and likewise, the presence of DNA strand breaks and abundant necrotic regions in tumors from PEITC-NAC fed mice are indicative of this mode of PCD [65]. Although molecular regulators of this proapoptotic response are not fully known, several biomarkers have been suggested in order to assess the proapoptotic effect of ITCs in future studies [12,92,154,159]. In this respect, it is important to underline the role of Bcl-2 and IAP family proteins, among others (Table 6).

The apoptotic response to ITCs in PCa cells is accompanied by a change in the ratio of proapoptotic–anti-apoptotic Bcl-2 family members. The levels of proapoptotic proteins Bad, Bak, Bax, and Bid are significantly upregulated in tumors of PEITC- or SFN-treated mice [99,150,152,156]. Additionally, the administration of these ITCs results in a marked decrease in the level of the antiapoptotic proteins Bcl-2 and Mcl-1 [152,154]. Besides Bcl-2 family proteins, the proapoptotic response to ITCs in PCa cells is associated with an altered expression of IAP family proteins, including XIAP and survivin [12,92,154]. It is known that IAP overexpression in tumors correlates with poor prognosis, aggressive disease and treatment resistance, and it is a strong predictor of human PCa recurrence because IAP proteins play a critical role in the regulation of PCD by inhibiting caspases [165]. Sakao *et al.* [92] have shown that oral gavage of PEITC is effective in suppressing XIAP expression, which is accompanied by the activation of caspase 3 [146], and cleavage and inactivation of PARP [65,146]. Recent evidences highlight the role of PARP in relation to androgen resistance and progression of PCa [166].

#### 5.2.3. Autophagy

Basal autophagy plays a critical role in maintaining cellular homeostasis and genome stability by removing exhausted, redundant or unwanted cellular components. In relation to cancer, this catabolic process acts by suppressing cell growth during the early stages of tumorigenesis [167] and it can be modulated by ITCs. Thus, for example, oral administration of PEITC at low micromolar concentrations arrests xenograft growth [157] and inhibits the progression of cancer by decreasing the expression of p62 (its overexpression correlates with an aggressive phenotype in prostate tumors) as well as the incidence and the size of poorly differentiated tumors [12]. Taking into account that the majority of PCa mortality is associated with an advanced disease, this fact is very important. The autophagic effect associated to PEITC is characterized by the accumulation of autophagosomes and increased expression of the microtubule-associated protein 1A/1B-light chain 3 (LC3) (Table 7) [12,157], which may serve as an endpoint to assess the biological activity of PEITC in future clinical studies.

### 5.3. Inhibition of Metastasis

Metastasis is a major cause of death in PCa patients [168]. Oral gavage of ITCs to TRAMP mice prevents pulmonary metastasis incidence and multiplicity [152], being the area occupied by the metastasis generally smaller compared with the area in control mice [12]. The pathogenesis of metastasis is complex and it is controlled by multiple molecules [169], which can be regulated by ITCs. In this respect, the influence of PEITC and/or SFN can be directed towards preventing the attachment of cancer cells to form tumors or they can act after the tumor is formed, as it is described below (Table 8).

On the one hand, the inhibitory effect of ITCs against metastasis can be dependent on the changes in E-cadherin expression, a suppressor of the invasion and growth of epithelial cancers because of its role in inhibition of epithelial to mesenchymal transition [170]. Dietary PEITC administration causes a statistically significant E-cadherin overexpression in the DLP of TRAMP mice [12], with a loss of expression of mesenchymal markers, such as vimentin [151].

On the other hand, the ITC-mediated inhibition of metastasis is associated with the blockage of the activation of NF-κB and its gene products, such as metalloproteinases, implicated in the degradation of the extracellular matrix and the promotion of tumor cell invasion and dissemination [170]. Shankar *et al.* [147] have observed that the treatment of tumor-bearing nude mice with SFN is effective in inhibiting MMP-2, MMP-7, MMP-9, MMP-14, TGF-β1 and uPAR expression. In relation to TGF-β1, it is important to highlight that it acts as a survival factor inhibiting chemotherapy-induced apoptosis in hormone-refractory PCa cells [171], which are suggested to possess cancer stem cell (CSC) characteristics [130]. CSC are believed to be a major cause of the resistance that cancer cells develop to drugs that initially shrink tumours [172], so CSC markers are a potential target for novel therapies against advanced PCa. Labsch *et al.* [123] have shown that the *in ovo* treatment of the PC3 xenograft tumors almost completely abolishes the expression of CSC markers, including CD133, CXCR4, Nanog, C-Met, EpCAM, CD44, ALDH1. Besides factors previously mentioned, other mediators regulated by ITCs which have been described to play a role in human PCa progression are integrin β6 [160], fibronectin 1 [160] and Notch 2 [129].

### 5.4. Inhibition of Angiogenesis

Angiogenesis plays a central role in the progression of hormone-refractory PCa. Microvessel density, a histological measure of tumor angiogenesis, correlates with Gleason score and predicts PCa progression [173]. Although available antiangiogenic therapy is showing hopeful results in advanced cancer, it is dose-limited due to adverse side effects [174], so new alternatives are necessary. In this context, it has been shown that ITCs can indirectly influence prostate tumor growth by microenvironment modulation, and specifically through altered angiogenesis. Hudson *et al.* [148] observed that treatment with PEITC inhibits the growth of LNCaP PCa cell xenografts in athymic nude mice without affecting cellular proliferation or apoptosis. It is thought that this effect is mediated by decreasing microvessel density, because this ITC inhibits the expression of the marker of angiogenesis PECAM-1/CD31 [148]. Likewise, PEITC alters morphology of the vessels [12], being considered as a sign of good prognosis [175]. The vessels appear to be more rounded and “regular” in shape in the DLP of TRAMP mice fed PEITC as opposed to the meandering and irregular vessels predominant in the DLP of control mice [12]. Besides PECAM-1/CD31, other mediators of angiogenesis whose expression can be modulated by ITC are shown in Table 9.

Angiogenesis suppression is not specific for one type of ITC, although these natural compounds may have different mechanisms of action. For example, SFN inhibits microvessel density by suppressing VEGF expression [147], whereas PEITC alters angiogenesis without affecting this growth factor [148].

Different *in vivo* experimental models can lead to distinct results in terms of efficacy of a particular ITC, without knowing, on occasions, the true motives of this incongruity. In this respect, it is known that markers of angiogenesis are suppressed when tumor-bearing nude mice are treated with SFN [147], although this ITC cannot inhibit angiogenesis in TRAMP mice [152].

## 6. Conclusions

PCa is a clinically heterogeneous disease (indolent, localized or invasive and metastatic) with multiple mechanisms and signaling pathways involved in its genesis and evolution, which could develop resistance to conventional treatment. As a result, new therapeutic approaches are required. It has been suggested that ITCs could have effect on different CaP cell populations, including CSCs and androgen-dependent and androgen-independent epithelial cells, both *in vitro* and *in vivo*. In this way, ITCs seem to be responsible for activating cell cycle arrest, apoptosis and autophagy. Likewise, they also seem to exhibit activity against metastasis and angiogenesis, acting on epigenetic mechanisms and different signaling pathways. Therefore, they offer an encouraging perspective for the research of new approaches in the chemoprevention and treatment of CaP. In spite of these findings, more studies are required because the effect of ITCs seems to be conditioned, among other factors, by the chemical structure of ITCS, the cell type and tumor stage.

## Figures and Tables

**Table 1:** Glucosinolates related to cancer prevention, single bioactive components after their hydrolysis classified into isothiocyanates, nitrile and indole compounds, and crucifer crops where these compounds are abundant. In this review, compounds highlighted in bold will be dealt with in detail.

Hydrolysis Products				
Glucosinolate	Isothiocyanates (ITCs)	Nitriles	Indoles	Crops or Species
**Aliphatic**				
Glucoraphanin	**Sulforaphane (SFN)**	Sulforaphane nitrile		Broccoli
Sinigrin	**Allyl isothiocyanate (AITC)**	Allyl nitrile		Kale, cabbage, Brussels sprouts, cauliflower
Glucoiberin	Iberin			Kale, cabbage, cauliflower
Glucoerucin	Erucin			Cabbage, broccoli
**Indolic**				
Glucobrassicin			Indole-3-carbinol (I3C)	Kale, cabbage, broccoli, Brussels sprouts, cauliflower
**Aromatic**				
Gluconasturtiin	**Phenethyl isothiocyanate (PEITC)**			Water cress (*Nasturtium officinalis*), white mustard (*Sinapis alba*), turnips
Glucotropaeolin	**Benzyl isothiocyanate (BITC)**			Indian cress or garden nasturtium or (*Tropaeolum majus*)

**Table 2:** Cell cycle arrest induced by ITCs *in vitro*.

ITC	Cells	Factor	Regulatory Partner(s)	Substrate	Effect	Ref.
AITC	LNCaP	↓ Cdk1	↓ Cyclin B1	↓ Cdc25B	G_2_/M phase arrest	[83]
				↓ Cdc25C		
AITC	PC3	↓ Cdk1	ns	↓ Cdc25B	G_2_/M phase arrest	[83]
				↓ Cdc25C		
BITC	DU 145	ns	↓ Cyclin B1	↓ Cdc25C	G_2_/M phase arrest	[91]
				↑ WEE1		
PEITC-NAC	LNCaP	↓ Cdk1	↓ Cyclin B1	ns	G_2_/M phase arrest	[85]
PEITC	LNCaP	↓ Cdk1	↓ Cyclin B1	ns	G_2_/M phase arrest	[85]
PEITC	PC3	↓ c-Myc	ns	↓ p21 mRNA and protein levels	G_0_/G_1_ phase arrest	[66]
PEITC	DU 145	↓ Cdk1	ns	↓ Cdc25C	G_2_/M phase arrest	[86]
				↑ p53		
				↑ WEE1		
PEITC	PC3	↓ Cdk1	ns	↓ Cdc25C	G_2_/M phase arrest	[84]
PEITC	LNCaP	↓ Cdk1	↓ Cyclin B1	ns	G_2_/M phase arrest	[85]
SFN-NAC	LNCaP	ns	↓ Cyclin D1	ns	G_1_ phase arrest	[95]
SFN	LNCaP	↓ Cdk1	↓ Cyclin B1	↓ Cdc25C	G_2_/M phase arrest	[88]
		ns	ns	↑ p21	G_2_/M phase arrest	[88]
				↑ p53		
		↓ Cdk4	ns	ns	S phase arrest	[88]
		↓ Cdk6	ns	ns	S phase arrest	[88]
SFN	BPH1, PC3	ns	ns	↑ p21 mRNA and protein levels	G_2_/M phase arrest	[96]
SFN	PrEC	ns	ns	≈ p21 mRNA protein levels	≈ G_2_/M phase	[96]

*Abbreviations*: ↑, enhanced expression or protein levels; ≈, no change in expression or protein levels; ↓, reduced expression or protein levels; AITC, allyl isothiocyanate; BITC, benzyl isothiocyanate; BPH1, benign hyperplasia epithelial cells; Cdc, cell division cycle proteins; Cdk, cyclin-dependent kinase; ns, not specified; p21, cyclin-dependent kinase inhibitor 1; PEITC, phenethyl isothiocyanate; PEITC-NAC, *N*-acetylcysteine conjugate of phenethyl isothiocyanate; PrEC, normal prostate epithelial cell line; SFN, sulforaphane; SFN-NAC, *N*-acetylcysteine conjugate of sulforaphane.

**Table 3:** Effects of ITCs on apoptosis *in vitro*.

ITC	Cells	Factor	Ref.
AITC	PC3, LNCaP	↓ Bcl-2	[83]
AITC	LNCaP	↓ Bcl-X_L_	[83]
BITC	PC3	↓ Bcl-2	[98]
PEITC	PC3	↓ Bcl-2, ↓ Bcl-X_L_ (+) caspase-8, caspase-9 pathways	[84]
PEITC	DU 145	(+) caspase-8-, caspase-9-, caspase-3 -dependent pathways	[86]
PEITC	LNCaP, PC3	(+) Bcl-2, (-) complex III activity	[92]
PEITC	PC3	↓ XIAP, ↓ survivin	[92]
PITC	DU 145	↓ Bcl-2, ↑ Bax (+) caspase 3	[86]
SFN	PC3	↑ Bax, ↓ Bcl-2, modified Bax:Bcl-2 ratio	[99]
SFN	DU 145	↓ Bcl-2	[99]
SFN	LNCaP, PC3	(+) Bax	[100]
SFN	PC3	↑ Apaf-1, (+) transcriptional E2F1	[100]
SFN	PC3	↓ Survivin	[101]
SFN	DU 145	(+) Caspase	[64]
SFN	PC3	(+) Caspase	[99]
SFN	PC3	↑ Bid, ↑ Smac/Diablo, ↑ ICAD, ↑ cytochrome c, ↑ c-IAP1, ↑ HSP27, ↑ Lamin A/C; ↑ BRE	[94]
SFN	PrEC, LNCaP, PC3	↑ Bax, ↑ MEK4, ↓ Lamin3	[94]
SFN	LNCaP	↓ Bim, ↓ Bmf	[94]
SFN	PrEC, PC3	↓ ASK1	[94]
SFN	PrEC	↓ cytochrome c, ↓ c-IAP1, ↓ HSP27	[94]
SFN	PC3, BPH1	(+) Multicaspase	[96]
SFN	BPH1	↓ HDAC2	[96]
SFN	BPH1, LNCaP, PC3	(−) HDAC, ↓ HDAC3, ↓ HDAC6	[96]
SFN	PC3	↓ Survivin	[101]
SFN	BPH1, LNCaP	↓ HDAC4	[96]
SFN	PC3	↓ Bid, ↓ Smac/Diablo, ↓ ICAD	[94]

*Abbreviations*: ↑, enhanced expression or protein levels; ↓, reduced expression or protein levels; (+), activation; (−), inhibition; AITC, allyl isothiocyanate; Apaf-1, apoptosis protease-activating factor-1; ASK 1, apoptosis signal-regulating kinase 1; Bax, Bcl-2-like protein 4; Bcl-2, B-cell lymphoma 2; Bid, BH3 interacting-domain death agonist; Bim, pro-apoptotic BH3-only protein; Bcl-X_L_, B-cell lymphoma-extra large; BITC, benzyl isothiocyanate; Bmf, Bcl-2-modifying factor; BPH1, benign hyperplasia epithelial cells; BRE, brain and rproductive organ-epressed protein; HDAC, histone deacetylases; HSP27, heat shock protein 27; IAP, inhibitor of apoptosis proteins; ICAD, inhibitor of caspase activated DNase; MEK4, mitogen-activated protein kinase 4; PEITC, phenethyl isothiocyanate; PITC, structural analogue of PEITC; PrEC, normal prostate epithelial cell line; SFN, sulforaphane; Smac/Diablo, second mitochondria-derived activator of caspases/Diablo homolog; XIAP, X-linked inhibitor of apoptosis protein.

**Table 4:** Discrepancies in the results between ITC studies in cultured prostate cancer cell lines and animal models.

ITC	Factor	*In Vitro*	*In Vivo*
PEITC	Bak	↑ [149]	↓ [150]
PEITC	Bcl-X_L_	↓ [84,149]	↑ [150]
PEITC	Vimentin	↑ [151]	↓ [151]
SFN	Bcl-X_L_	≈ [99]	↓ [147]
SFN	Bid	≈ [99]	↑ [152]

*Abbreviations*: ↑, enhanced expression; ≈, no change in expression; ↓, reduced expression; Bak, Bcl-2 homologous antagonist/killer; Bcl-X_L_, B-cell lymphoma-extra large; Bid, BH3 interacting-domain death agonist; PEITC, phenethyl isothiocyanate; SFN, sulforaphane.

**Table 5:** ITCs inhibit the *in vivo* and *ex vivo* growth of prostate tumors by inhibiting tumor cell proliferation.

ITC	Factor	Model (Cell Line)	Ref.
BITC	↓ Ki-67	TRAMP	[161]
BITC	↓ Cyclin D1	TRAMP	[161]
BITC	↓ Cyclin A	TRAMP	[161]
BITC	↓ Cdk2	TRAMP	[161]
PEITC	↓ Akt	TRAMP	[155]
PEITC	↓ FKHR	TRAMP	[155]
PEITC	↑ IGFBP3	Subcutaneous xenograft (LNCaP)	[160]
PEITC	≈ Ki-67	Subcutaneous xenograft (LNCaP)	[148]
PEITC	↓ Ki-67	TRAMP	[12]
PEITC	≈ PCNA	Subcutaneous xenograft (LNCaP)	[148]
PEITC	↓ PCNA	TRAMP	[155]
PEITC	↓ PCNA	Subcutaneous xenograft (PC3)	[154]
PEITC	↓ PDK1	TRAMP	[155]
PEITC-NAC	↓ Cyclin D1	Subcutaneous xenograft (PC3)	[65]
PEITC-NAC	↓ Cyclin E	Subcutaneous xenograft (PC3)	[65]
PEITC-NAC	↑ p21	Subcutaneous xenograft (PC3)	[65]
PEITC-NAC	↑ p27	Subcutaneous xenograft (PC3)	[65]
PEITC-NAC	↓ pRb	Subcutaneous xenograft (PC3)	[65]
SFN	↓ COX-2	Orthotopic assay (PC3)	[147]
SFN	↓ Cyclin D1	Orthotopic assay (PC3)	[147]
SFN	↓ IL-6	Orthotopic assay (PC3)	[147]
SFN	↓ IL-8	Orthotopic assay (PC3)	[147]
SFN	↓ Ki-67	Orthotopic assay (PC3)	[147]
SFN	↓ Ki-67	CAM xenograft (PC3)	[123]
SFN	↓ PCNA	TRAMP	[152]
SFN	↓ PCNA	Orthotopic assay (PC3)	[147]

*Abbreviations*: ↑, enhanced expression; ≈, no change in expression; ↓, reduced expression; BITC, benzyl isothiocyanate; CAM, chorioallantoic membrane; COX-2, cyclooxygenase-2; FKHR, forkhead transcription factor FOXO1; IGFBP3, insulin-like growth factor binding protein 3; IL, interleukin; p21, cyclin-dependent kinase inhibitor 1; p27, cyclin-dependent kinase inhibitor 1B; PCNA, proliferating cell nuclear antigen; PDK1, pyruvate dhydrogenase kinase, iozyme 1; PEITC, phenethyl isothiocyanate; PEITC-NAC, *N*-acetylcysteine conjugate of phenethyl isothiocyanate; SFN, sulforaphane; pRb, Rb protein; TRAMP, transgenic adenocarcinoma of the mouse prostate model.

**Table 6:** ITCs inhibit the *in vivo* and *ex vivo* growth of prostate tumors by inducing apoptosis.

ITC	Factor	Model (Cell Line)	Ref.
AITC	≈ Bax	Subcutaneous xenograft (PC3)	[162]
AITC	↓ Bcl-2	Subcutaneous xenograft (PC3)	[162]
AITC	≈ Bcl-X_L_	Subcutaneous xenograft (PC3)	[162]
AITC	↑ Bid	Subcutaneous xenograft (PC3)	[162]
AITC	≈ Clusterin	TRAMP	[12]
PEITC	↑ Bad	TRAMP	[155]
PEITC	≈ Bad	Subcutaneous xenograft (PC3)	[146]
PEITC	↓ Bak	Subcutaneous xenograft (PC3)	[150]
PEITC	≈ Bak	Subcutaneous xenograft (PC3)	[154]
PEITC	↑ Bax	Subcutaneous xenograft (PC3)	[150]
PEITC	≈ Bax	Subcutaneous xenograft (LNCaP)	[148]
PEITC	≈ Bax	Subcutaneous xenograft (PC3)	[154]
PEITC	↓ Bcl-2	Subcutaneous xenograft (PC3)	[154]
PEITC	↑ Bcl-X_L_	Subcutaneous xenograft (PC3)	[150]
PEITC	↑ Bid	Subcutaneous xenograft (PC3)	[150]
PEITC	↓ Bok	Subcutaneous xenograft (PC3)	[150]
PEITC	≈ Caspasa 3	Subcutaneous xenograft (LNCaP)	[148]
PEITC	↑ Caspase-3	TRAMP	[155]
PEITC	↑ Caspase-3	Subcutaneous xenograft (PC3)	[146]
PEITC	↓ Clusterin	TRAMP	[12]
PEITC	↓ GSK3βα	Subcutaneous xenograft (PC3)	[146]
PEITC	↓ IκBα	Subcutaneous xenograft (PC3)	[146]
PEITC	↓ IKKβα	Subcutaneous xenograft (PC3)	[146]
PEITC	≈ p66^Shc^	Subcutaneous xenograft (PC3)	[159]
PEITC	↑ PARP	Subcutaneous xenograft (PC3)	[146]
PEITC	↓ Pin1	Subcutaneous xenograft (PC3)	[159]
PEITC	↑ RANBP1	Subcutaneous xenograft (LNCaP)	[160]
PEITC	↓ Survivin	TRAMP	[92]
PEITC	↓ XIAP	TRAMP	[92]
PEITC	≈ XIAP	Subcutaneous xenograft (PC3)	[154]
PEITC-NAC	↑ PARP	Subcutaneous xenograft (PC3)	[65]
SFN	↑ Bad	TRAMP	[152]
SFN	↑ Bak	Orthotopic assay (PC3)	[147]
SFN	↑ Bak	TRAMP	[152]
SFN	↑ Bax	Orthotopic assay (PC3)	[147]
SFN	↑ Bax	Subcutaneous xenograft (PC3)	[99,156]
SFN	↑ Bax	TRAMP	[152]
SFN	↓ Bcl-2	Orthotopic assay (PC3)	[147]
SFN	↓ Bcl-2	Subcutaneous xenograft (PC3)	[99]
SFN	≈ Bcl-X_L_	Subcutaneous xenograft (PC3)	[99]
SFN	↓ Bcl-X_L_	Orthotopic assay (PC3)	[147]
SFN	↑ Bid	Subcutaneous xenograft (PC3)	[99]
SFN	↑ Bid	TRAMP	[152]
SFN	↑ Caspase 3	CAM xenograft (PC3)	[123]
SFN	↑ Caspase-3	Orthotopic assay (PC3)	[147]
SFN	↑ Caspase-8	Orthotopic assay (PC3)	[147]
SFN	↓ Clusterin	TRAMP	[12]
SFN	↓ Mcl-1	TRAMP	[152]
SFN	↑ PARP	TRAMP	[152]

*Abbreviations*: ↑, enhanced expression; ≈, no change in expression; ↓, reduced expression; AITC, allyl isothiocyanate; Bad, Bcl-2-associated death promoter; Bak, Bcl-2 homologous antagonist/killer; Bax, Bcl-2-like protein 4; Bcl-2, B-cell lymphoma 2; Bcl-X_L_, B-cell lymphoma-extra large; Bid, BH3 interacting-domain death agonist; Bok, Bcl-2 related ovarian killer; CAM, chorioallantoic membrane; GSK3, gycogen synthase kinase 3; IκBα, nuclear factor of kappa light polypeptide gene enhancer in B-cells inhibitor, alpha; IKKβα, IκB kinase beta alpha; Mcl-1, myeloid cell leukemia 1; p66^Shc^, 66-kDa Src collagen homologue (Shc) adaptor protein; PARP, poly(ADP-ribose) polymerase; PEITC, phenethyl isothiocyanate; PEITC-NAC, *N*-acetylcysteine conjugate of phenethyl isothiocyanate; Pin1, prolyl isomerase; RANBP1, ras-related nuclear protein (RAN) binding protein 1; SFN, sulforaphane; TRAMP, transgenic adenocarcinoma of the mouse prostate model; XIAP, X-linked inhibitor of apoptosis.

**Table 7:** ITCs inhibit the *in vivo* growth of prostate tumors by inducing autophagic cell death.

ITC	Factor	Model (Cell Line)	Ref.
PEITC	↑ LC3	Subcutaneous xenograft (PC3)	[157]
PEITC	↑ LC3	TRAMP	[12]
PEITC	↓ p62	TRAMP	[12]

*Abbreviations*: ↑, enhanced expression; ↓, reduced expression; LC3, microtubule-associated protein 1 light chain 3; p62, sequestosome 1 or p62/SQSTM1; PEITC, phenethyl isothiocyanate; TRAMP, transgenic adenocarcinoma of the mouse prostate model.

**Table 8:** ITCs inhibit the *in vivo* and *ex vivo* growth of prostate tumors by inhibiting invasion and metastasis.

ITC	Factor	Model (Cell Line)	Reference
PEITC	↑ E-cadherin	TRAMP	[12]
PEITC	↑ Fibronectin 1	Subcutaneous xenograft (LNCaP)	[160]
PEITC	↓ Integrin β6	Subcutaneous xenograft (LNCaP)	[160]
PEITC	↑ Notch2	Subcutaneous xenograft (PC3)	[129]
PEITC	↑ Notch2	TRAMP	[129]
PEITC	↓ Vimentin	TRAMP	[151]
PEITC	↓ CSC markers *	Subcutaneous xenograft (PC3)	[122]
SFN	↓ CSC markers *	CAM xenograft (PC3)	[123]
SFN	≈ E-cadherin	TRAMP	[152]
SFN	↓ MMP-2	Orthotopic assay (PC3)	[147]
SFN	↓ MMP-7	Orthotopic assay (PC3)	[147]
SFN	↓ MMP-9	Orthotopic assay (PC3)	[147]
SFN	↓ MMP-14	Orthotopic assay (PC3)	[147]
SFN	↓ NF-κB	Orthotopic assay (PC3)	[147]
SFN	↓ TGF-β1	Orthotopic assay (PC3)	[147]
SFN	↓ uPAR	Orthotopic assay (PC3)	[147]

*Abbreviations*: ↑, enhanced expression; ≈, no change in expression; ↓, reduced expression; CAM, chorioallantoic membrane; CSC: cancer stem cells; MMP, matrix metalloproteinase; NF-κB, nuclear factor kappa-light-chain-enhancer of activated B cells; Notch 2, neurogenic locus notch homolog protein 2; PEITC, phenethyl isothiocyanate; SFN, sulforaphane; TGF-β1, transforming growth factor-β1; TRAMP, transgenic adenocarcinoma of the mouse prostate model; uPAR, urokinase-type plasminogen activator receptor. * CD133, CXCR4, Nanog, C-Met, EpCAM, CD44, or ALDH1.

**Table 9:** ITCs inhibit the *in vivo* growth of prostate tumors by inhibiting angiogenesis.

ITC	Factor	Model (Cell Line)	Reference
PEITC	≈ PECAM-1/CD31	TRAMP	[12]
PEITC	↓ PECAM-1/CD31	Subcutaneous xenograft (LNCaP)	[148]
PEITC	≈ VEGF	Subcutaneous xenograft (LNCaP)	[148]
SFN	≈ PECAM-1/CD31	TRAMP	[152]
SFN	↓ Akt	Orthotopic assay (PC3)	[147]
SFN	↓ ERK1/2	Orthotopic assay (PC3)	[147]
SFN	↓ FOXO3a	Orthotopic assay (PC3)	[147]
SFN	↓ HIF-1 α	Orthotopic assay (PC3)	[147]
SFN	↓ IL-6	Orthotopic assay (PC3)	[147]
SFN	↓ IL-8	Orthotopic assay (PC3)	[147]
SFN	↓ TGF-β1	Orthotopic assay (PC3)	[147]
SFN	↓ VEGF	Orthotopic assay (PC3)	[147]

*Abbreviations*: ≈, no change in expression; ↓, reduced expression; ERK1/2, extracellular signal-regulated kinase 1/2; FOXO3a, forkhead box O3; HIF-1α, hypoxia-inducible factor 1-alpha; IL, interleukin; PECAM-1/CD31, tumor platelet/endothelial cell adhesion molecule; PEITC, phenethyl isothiocyanate; SFN, sulforaphane; TGF-β1, transforming growth factor-β1; TRAMP, transgenic adenocarcinoma of the mouse prostate model; VEGF, vascular endothelial growth factor.

## References

[1] Higdon J.V., Delage B., Williams D.E., Dashwood R.H. (2007). Cruciferous vegetables and human cancer risk: Epidemiologic evidence and mechanistic basis. Pharmacol. Res..

[2] Herr I., Büchler M.W. (2010). Dietary constituents of broccoli and other cruciferous vegetables: Implications for prevention and therapy of cancer. Cancer Treat. Rev..

[3] Fenwick G.R., Heaney R.K., Mullin W.J. (1983). Glucosinolates and their breakdown products in food and food plants. Crit. Rev. Food Sci. Nutr..

[4] Cartea M.E., Rodríguez V.M., de Haro A., Velasco P., Ordás A. (2008). Variation of glucosinolates and nutritional value in nabicol (Brassica napus pabularia group). Euphytica.

[5] Cartea M.E., de Haro A., Obregón S., Soengas P., Velasco P. (2012). Glucosinolate variation in leaves of Brassica rapa crops. Plant Foods Hum. Nutr..

[6] Fahey J.W., Zalcmann A.T., Talalay P. (2001). The chemical diversity and distribution of glucosinolates and isothiocyanates among plants. Phytochemistry.

[7] Mithen R.F., Dekker M., Verkerk R., Rabot S., Johnson I.T. (2000). The nutritional significance, biosynthesis and bioavailability of glucosinolates in human foods. J. Sci. Food Agric..

[8] Talalay P., Fahey J.W. (2001). Phytochemicals from cruciferous plants protect against cancer by modulating carcinogen metabolism. J. Nutr..

[9] Traka M., Mithen R.F. (2009). Glucosinolates, isothiocyanates and human health. Phytochem. Rev..

[10] Manchali S., Murthy K.N.C., Patil B.S. (2012). Crucial facts about health benefits of popular cruciferous vegetables. J. Funct. Foods.

[11] Wattenberg L.W. (1977). Inhibition of carcinogenic effects of polycyclic hydrocarbons by benzyl isothiocyanate and related compounds. J. Natl. Cancer Inst..

[12] Powolny A.A., Bommareddy A., Hahm E.R., Normolle D.P., Beumer J.H., Nelson J.B., Singh S.V. (2011). Chemopreventative potential of the cruciferous vegetable constituent phenethyl isothiocyanate in a mouse model of prostate cancer. J. Natl. Cancer Inst..

[13] Gupta P., Srivastava S.K. (2012). Antitumor activity of phenethyl isothiocyanate in HER2-positive breast cancer models. BMC Med..

[14] Verhoeven D.T., Goldbohm R.A., van Poppel G., Verhagen H., van den Brandt P.A. (1996). Epidemiological studies on brassica vegetables and cancer risk. Cancer Epidemiol. Biomark. Prev..

[15] Graham S., Dayal H., Swanson M., Mittelman A., Wilkinson G. (1978). Diet in the epidemiology of cancer of the colon and rectum. J. Natl. Cancer Inst..

[16] Gupta P., Kim B., Kim S.H., Srivastava S.K. (2014). Molecular targets of isothiocyanates in cancer: Recent advances. Mol. Nutr. Food Res..

[17] Kirsh V.A., Peters U., Mayne S.T., Subar A.F., Chatterjee N., Johnson C.C., Hayes R.B. (2007). Prostate, Lung, Colorectal and Ovarian Cancer Screening Trial. Prospective study of fruit and vegetable intake and risk of prostate cancer. J. Natl. Cancer Inst..

[18] Juge N., Mithen R.F., Traka M. (2007). Molecular basis for chemoprevention by sulforaphane: A comprehensive review. Cell Mol. Life Sci..

[19] Fuentes F., Paredes-Gonzalez X., Kong A.T. (2015). Dietary glucosinolates sulforaphane, phenethyl isothiocyanate, indole-3-carbinol/3,3′-diindolylmethane: Anti-oxidative stress/inflammation, Nrf2, epigenetics/epigenomics and *in vivo* cancer chemopreventive efficacy. Curr. Pharmacol. Rep..

[20] Valgimigli L., Iori R. (2009). Antioxidant and pro-oxidant capacities of ITCs. Environ. Mol. Mutagen..

[21] Dworkin A.M., Huang T.H., Toland A.E. (2009). Epigenetic alterations in the breast: Implications for breast cancer detection, prognosis and treatment. Semin. Cancer Biol..

[22] Basse C., Arock M. (2015). The increasing roles of epigenetics in breast cancer: Implications for pathogenicity, biomarkers, prevention and treatment. Int. J. Cancer.

[23] Berger S.L., Kouzarides T., Shiekhattar R., Shilatifard A. (2009). An operational definition of epigenetics. Genes Dev..

[24] Sharma S., Kelly T.K., Jones P.A. (2010). Epigenetics in cancer. Carcinogenesis.

[25] Nowsheen S., Aziz K., Tran P.T., Gorgoulis V.G., Yang E.S., Georgakilas A.G. (2014). Epigenetic inactivation of DNA repair in breast cancer. Cancer Lett..

[26] Ferguson L.R., Chen H., Collins A.R., Connell M., Damia G., Dasgupta S., Malhotra M., Meeker A.K., Amedei A., Amin A. (2015). Genomic instability in human cancer: Molecular insights and opportunities for therapeutic attack and prevention through diet and nutrition. Semin. Cancer Biol..

[27] Wong C.P., Hsu A., Buchanan A., Palomera-Sanchez Z., Beaver L.M., Houseman E.A., Williams D.E., Dashwood R.H., Ho E. (2014). Effects of sulforaphane and 3,3′-diindolylmethane on genome-wide promoter methylation in normal prostate epithelial cells and prostate cancer cells. PLoS ONE.

[28] Denis H., Ndlovu M.N., Fuks F. (2011). Regulation of mammalian DNA methyltransferases: A route to new mechanisms. EMBO Rep..

[29] Morey Kinney S.R., Smiraglia D.J., James S.R., Moser M.T., Foster B.A., Karpf A.R. (2008). Stage-specific alterations of DNA methyltransferase expression, DNA hypermethylation, and DNA hypomethylation during prostate cancer progression in the transgenic adenocarcinoma of mouse prostate model. Mol. Cancer Res..

[30] Lin R.K., Wu C.Y., Chang J.W., Juan L.J., Hsu H.S., Chen C.Y., Lu Y.Y., Tang Y.A., Yang Y.C., Yang P.C. (2010). Dysregulation of p53/Sp1 control leads to DNA methyltransferase-1 overexpression in lung cancer. Cancer Res..

[31] Mizuno S., Chijiwa T., Okamura T., Akashi K., Fukumaki Y., Niho Y., Sasaki H. (2001). Expression of DNA methyltransferases DNMT1, 3A, and 3B in normal hematopoiesis and in acute and chronic myelogenous leukemia. Blood.

[32] He S., Wang F., Yang L., Guo C., Wan R., Ke A., Xu L., Hu G., Xu X., Shen J. (2011). Expression of DNMT1 and DNMT3a are regulated by GLI1 in human pancreatic cancer. PLoS ONE.

[33] Etoh T., Kanai Y., Ushijima S., Nakagawa T., Nakanishi Y., Sasako M., Kitano S., Hirohashi S. (2004). Increased DNA methyltransferase 1 (DNMT1) protein expression correlates significantly with poorer tumor differentiation and frequent DNA hypermethylation of multiple CpG islands in gastric cancers. Am. J. Pathol..

[34] Sproul D., Gilbert N., Bickmore W.A. (2005). The role of chromatin structure in regulating the expression of clustered genes. Nat. Rev. Genet..

[35] Kouzarides T. (2007). Chromatin modifications and their function. Cell.

[36] Cedar H., Bergman Y. (2009). Linking DNA methylation and histone modification: Patterns and paradigms. Nat. Rev. Genet..

[37] Turner B.M. (2000). Histone acetylation and an epigenetic code. Bioessays.

[38] Sarkar S., Horn G., Moulton K., Oza A., Byler S., Kokolus S., Longacre M. (2013). Cancer development, progression, and therapy: An epigenetic overview. Int. J. Mol. Sci..

[39] Byler S., Sarkar S. (2014). Do epigenetic drug treatments hold the key to killing cancer progenitor cells?. Epigenomics.

[40] Ong T.P., Moreno F.S., Ross S.A. (2011). Targeting the epigenome with bioactive food components for cancer prevention. J. Nutrigenet. Nutrigenom..

[41] Khan S.I., Aumsuwan P., Khan I.A., Walker L.A., Dasmahapatra A.K. (2012). Epigenetic events associated with breast cancer and their prevention by dietary components targeting the epigenome. Chem. Res. Toxicol..

[42] Royston K.J., Tollefsbol T.O. (2015). The epigenetic impact of cruciferous vegetables on cancer prevention. Curr. Pharmacol. Rep..

[43] Meeran S.M., Patel S.N., Li Y., Shukla S., Tollefsbol T.O. (2012). Bioactive dietary supplements reactivate ER expression in ER-negative breast cancer cells by active chromatin modifications. PLoS ONE.

[44] Gerhauser C. (2013). Epigenetic impact of dietary isothiocyanates in cancer chemoprevention. Curr. Opin. Clin. Nutr. Metab. Care.

[45] Singh S.V., Singh K. (2012). Cancer chemoprevention with dietary isothiocyanates mature for clinical translational research. Carcinogenesis.

[46] Forster T., Rausch V., Zhang Y., Isayev O., Heilmann K., Schoensiegel F., Liu L., Nessling M., Richter K., Labsch S. (2014). Sulforaphane counteracts aggressiveness of pancreatic cancer driven by dysregulated Cx43-mediated gap junctional intercellular communication. Oncotarget.

[47] Rajendran P., Kidane A.I., Yu T.W., Dashwood W.M., Bisson W.H., Löhr C.V., Ho E., Williams D.E., Dashwood R.H. (2013). HDAC turnover, CtIP acetylation and dysregulated DNA damage signaling in colon cancer cells treated with sulforaphane and related dietary isothiocyanates. Epigenetics.

[48] Balasubramanian S., Chew Y.C., Eckert R.L. (2011). Sulforaphane suppresses polycomb group protein level via a proteasome-dependent mechanism in skin cancer cells. Mol. Pharmacol..

[49] Chew Y.C., Adhikary G., Wilson G.M., Xu W., Eckert R.L. (2012). Sulforaphane induction of p21(Cip1) cyclin-dependent kinase inhibitor expression requires p53 and Sp1 transcription factors and is p53-dependent. J. Biol. Chem..

[50] Su Z.Y., Zhang C., Lee J.H., Shu L., Wu T.Y., Khor T.O., Conney A.H., Lu Y.P., Kong A.N. (2014). Requirement and epigenetics reprogramming of Nrf2 in suppression of tumor promoter TPA-induced mouse skin cell transformation by sulforaphane. Cancer Prev. Res..

[51] Wang L.G., Chiao J.W. (2010). Prostate cancer chemopreventive activity of phenethyl isothiocyanate through epigenetic regulation (review). Int. J. Oncol..

[52] Traka M.H., Melchini A., Mithen R.F. (2014). Sulforaphane and prostate cancer interception. Drug Discov. Today.

[53] Hsu A., Wong C.P., Yu Z., Williams D.E., Dashwood R.H., Ho E. (2011). Promoter de-methylation of cyclin D2 by sulforaphane in prostate cancer cells. Clin. Epigenet..

[54] Meeran S.M., Patel S.N., Tollefsbol T.O. (2010). Sulforaphane causes epigenetic repression of hTERT expression in human breast cancer cell lines. PLoS ONE.

[55] Myzak M.C., Hardin K., Wang R., Dashwood R.H., Ho E. (2006). Sulforaphane inhibits histone deacetylase activity in BPH-1, LnCaP and PC-3 prostate epithelial cells. Carcinogenesis.

[56] Myzak M.C., Dashwood W.M., Orner G.A., Ho E., Dashwood R.H. (2006). Sulforaphane inhibits histone deacetylase *in vivo* and suppresses tumorigenesis in Apc-minus mice. FASEB J..

[57] Marks P., Rifkind R.A., Richon V.M., Breslow R., Miller T., Kelly W.K. (2001). Histone deacetylases and cancer: Causes and therapies. Nat. Rev. Cancer.

[58] Gibbs A., Schwartzman J., Deng V., Alumkal J. (2009). Sulforaphane destabilizes the androgen receptor in prostate cancer cells by inactivating histone deacetylase 6. Proc. Natl. Acad. Sci. USA.

[59] Watson P.A., Arora V.K., Sawyers C.L. (2015). Emerging mechanisms of resistance to androgen receptor inhibitors in prostate cancer. Nat. Rev. Cancer..

[60] Huggins C., Stevens R.E., Hodges C.V. (1941). Studies on prostatic cancer. II. The effects of castration on advanced carcinoma of the prostate gland. Arch. Surg..

[61] Marrocco D.L., Tilley W.D., Bianco-Miotto T., Evdokiou A., Scher H.I., Rifkind R.A., Marks P.A., Richon V.M., Butler L.M. (2007). Suberoylanilide hydroxamic acid (vorinostat) represses androgen receptor expression and acts synergistically with an androgen receptor antagonist to inhibit prostate cancer cell proliferation. Mol. Cancer Ther..

[62] Chen L., Meng S., Wang H., Bali P., Bai W., Li B., Atadja P., Bhalla K.N., Wu J. (2005). Chemical ablation of androgen receptor in prostate cancer cells by the histone deacetylase inhibitor LAQ824. Mol. Cancer Ther..

[63] Wang L.G., Liu X.M., Chiao J.W. (2006). Repression of androgen receptor in prostate cancer cells by phenethyl isothiocyanate. Carcinogenesis.

[64] Wang L., Liu D., Ahmed T., Chung F.L., Conaway C., Chiao J.W. (2004). Targeting cell cycle machinery as a molecular mechanism of sulforaphane in prostate cancer prevention. Int. J. Oncol..

[65] Chiao J.W., Wu H., Ramaswamy G., Conaway C.C., Chung F.L., Wang L., Liu D. (2004). Ingestion of an isothiocyanate metabolite from cruciferous vegetables inhibits growth of human prostate cancer cell xenografts by apoptosis and cell cycle arrest. Carcinogenesis.

[66] Wang L.G., Liu X.M., Fang Y., Dai W., Chiao F.B., Puccio G.M., Feng J., Liu D., Chiao J.W. (2008). De-repression of the p21 promoter in prostate cancer cells by an isothiocyanate via inhibition of HDACs and c-Myc. Int. J. Oncol..

[67] Maruyama R., Toyooka S., Toyooka K.O., Virmani A.K., Zöchbauer-Müller S., Farinas A.J., Minna J.D., McConnell J., Frenkel E.P., Gazdar A.F. (2002). Aberrant promoter methylation profile of prostate cancers and its relationship to clinicopathological features. Clin. Cancer Res..

[68] Woodson K., Gillespie J., Hanson J., Emmert-Buck M., Phillips J.M., Linehan W.M., Tangrea J.A. (2004). Heterogeneous gene methylation patterns among pre-invasive and cancerous lesions of the prostate: A histopathologic study of whole mount prostate specimens. Prostate.

[69] Wang L.G., Beklemisheva A., Liu X.M., Ferrari A.C., Feng J., Chiao J.W. (2007). Dual action on promoter demethylation and chromatin by an isothiocyanate restored GSTP1 silenced in prostate cancer. Mol. Carcinog..

[70] Dasgupta S., Srinidhi S., Vishwanatha J.K. (2012). Oncogenic activation in prostate cancer progression and metastasis: Molecular insights and future challenges. J. Carcinog..

[71] Cunningham D., You Z. (2015). *In vitro* and *in vivo* model systems used in prostate cancer research. J. Biol. Methods.

[72] Simone C.B., John-Aryankalayil M., Palayoor S.T., Makinde A.Y., Cerna D., Falduto M.T., Magnuson S.R., Coleman C.N. (2013). mRNA expression profiles for prostate cancer following fractionated irradiation are influenced by p53 status. Transl. Oncol..

[73] Saha A., Blando J., Silver E., Beltran L., Sessler J., DiGiovanni J. (2014). 6-Shogaol from dried ginger inhibits growth of prostate cancer cells both *in vitro* and *in vivo* through inhibition of STAT3 and NF-κB signaling. Cancer Prev. Res..

[74] Perry J.A., Kornbluth S. (2007). Cdc25 and Wee1: Analogous opposites?. Cell Div..

[75] Clarke J.D., Dashwood R.H., Ho E. (2008). Multi-targeted prevention of cancer by sulforaphane. Cancer Lett..

[76] De Witt Hamer P.C., Mir S.E., Noske D., van Noorden C.J., Würdinger T. (2011). WEE1 kinase targeting combined with DNA-damaging cancer therapy catalyzes mitotic catastrophe. Clin. Cancer Res..

[77] Ngan E.S., Hashimoto Y., Ma Z.Q., Tsai M.J., Tsai S.Y. (2003). Overexpression of Cdc25B, an androgen receptor coactivator, in prostate cancer. Oncogene.

[78] Ozen M., Ittmann M. (2005). Increased expression and activity of CDC25C phosphatase and an alternatively spliced variant in prostate cancer. Clin. Cancer Res..

[79] Chiu Y.T., Han H.Y., Leung S.C., Yuen H.F., Chau C.W., Guo Z., Qiu Y., Chan K.W., Wang X., Wong Y.C. (2009). CDC25A functions as a novel Ar corepressor in prostate cancer cells. J. Mol. Biol..

[80] Mirzayans R., Andrais B., Scott A., Wang Y.W., Murray D. (2013). Ionizing radiation-induced responses in human cells with differing TP53 status. Int. J. Mol. Sci..

[81] Fizazi K., Martinez L.A., Sikes C.R., Johnston D.A., Stephens L.C., McDonnell T.J., Logothetis C.J., Trapman J., Pisters L.L., Ordoñez N.G. (2002). The association of p21((WAF-1/CIP1)) with progression to androgen-independent prostate cancer. Clin. Cancer Res..

[82] De Luca P., Moiola C.P., Zalazar F., Gardner K., Vazquez E.S., de Siervi A. (2013). BRCA1 and p53 regulate critical prostate cancer pathways. Prostate Cancer Prostatic. Dis..

[83] Xiao D., Srivastava S.K., Lew K.L., Zeng Y., Hershberger P., Johnson C.S., Trump D.L., Singh S.V. (2003). Allyl isothiocyanate, a constituent of cruciferous vegetables, inhibits proliferation of human prostate cancer cells by causing G2/M arrest and inducing apoptosis. Carcinogenesis.

[84] Xiao D., Johnson C.S., Trump D.L., Singh S.V. (2004). Proteasome-mediated degradation of cell division cycle 25C and cyclin-dependent kinase 1 in phenethyl isothiocyanate-induced G2-M-phase cell cycle arrest in PC-3 human prostate cancer cells. Mol. Cancer Ther..

[85] Hwang E.S., Lee H.J. (2010). Effects of phenylethyl isothiocyanate and its metabolite on cell-cycle arrest and apoptosis in LNCaP human prostate cancer cells. Int. J. Food Sci. Nutr..

[86] Tang N.Y., Huang Y.T., Yu C.S., Ko Y.C., Wu S.H., Ji B.C., Yang J.S., Yang J.L., Hsia T.C., Chen Y.Y. (2011). Phenethyl isothiocyanate (PEITC) promotes G2/M phase arrest via p53 expression and induces apoptosis through caspase- and mitochondria-dependent signaling pathways in human prostate cancer DU 145 cells. Anticancer Res..

[87] Qu Y., Oyan A.M., Liu R., Hua Y., Zhang J., Hovland R., Popa M., Liu X., Brokstad K.A., Simon R. (2013). Generation of prostate tumor-initiating cells is associated with elevation of reactive oxygen species and IL-6/STAT3 signaling. Cancer Res..

[88] Herman-Antosiewicz A., Xiao H., Lew K.L., Singh S.V. (2007). Induction of p21 protein protects against sulforaphane-induced mitotic arrest in LNCaP human prostate cancer cell line. Mol. Cancer Ther..

[89] Singh S.V., Herman-Antosiewicz A., Singh A.V., Lew K.L., Srivastava S.K., Kamath R., Brown K.D., Zhang L., Baskaran R. (2004). Sulforaphane-induced G2/M phase cell cycle arrest involves checkpoint kinase 2-mediated phosphorylation of cell division cycle 25C. J. Biol. Chem..

[90] Cho S.D., Li G., Hu H., Jiang C., Kang K.S., Lee Y.S., Kim S.H., Lu J. (2005). Involvement of c-Jun *N*-terminal kinase in G2/M arrest and caspase-mediated apoptosis induced by sulforaphane in DU145 prostate cancer cells. Nutr. Cancer..

[91] Liu K.C., Huang Y.T., Wu P.P., Ji B.C., Yang J.S., Yang J.L., Chiu T.H., Chueh F.S., Chung J.G. (2011). The roles of AIF and Endo G in the apoptotic effects of benzyl isothiocyanate on DU 145 human prostate cancer cells via the mitochondrial signaling pathway. Int. J. Oncol..

[92] Sakao K., Desineni S., Hahm E.R., Singh S.V. (2012). Phenethyl isothiocyanate suppresses inhibitor of apoptosis family protein expression in prostate cancer cells in culture and *in vivo*. Prostate.

[93] Gong A., He M., Krishna Vanaja D., Yin P., Karnes R.J., Young C.Y. (2009). Phenethyl isothiocyanate inhibits STAT3 activation in prostate cancer cells. Mol. Nutr. Food Res..

[94] Beaver L.M., Buchanan A., Sokolowski E.I., Riscoe A.N., Wong C.P., Chang J.H., Löhr C.V., Williams D.E., Dashwood R.H., Ho E. (2014). Transcriptome analysis reveals a dynamic and differential transcriptional response to sulforaphane in normal and prostate cancer cells and suggests a role for Sp1 in chemoprevention. Mol. Nutr. Food Res..

[95] Chiao J.W., Chung F.L., Kancherla R., Ahmed T., Mittelman A., Conaway C.C. (2002). Sulforaphane and its metabolite mediate growth arrest and apoptosis in human prostate cancer cells. Int. J. Oncol..

[96] Clarke J.D., Hsu A., Yu Z., Dashwood R.H., Ho E. (2011). Differential effects of sulforaphane on histone deacetylases, cell cycle arrest and apoptosis in normal prostate cells *vs.* hyperplastic and cancerous prostate cells. Mol. Nutr. Food Res..

[97] Portt L., Norman G., Clapp C., Greenwood M., Greenwood M.T. (2011). Anti-apoptosis and cell survival: A review. Biochim. Biophys. Acta.

[98] Lin J.F., Tsai T.F., Liao P.C., Lin Y.H., Lin Y.C., Chen H.E., Chou K.Y., Hwang T.I. (2013). Benzyl isothiocyanate induces protective autophagy in human prostate cancer cells via inhibition of mTOR signaling. Carcinogenesis.

[99] Singh A.V., Xiao D., Lew K.L., Dhir R., Singh S.V. (2004). Sulforaphane induces caspase-mediated apoptosis in cultured PC-3 human prostate cancer cells and retards growth of PC-3 xenografts *in vivo*. Carcinogenesis.

[100] Choi S., Lew K.L., Xiao H., Herman-Antosiewicz A., Xiao D., Brown C.K., Singh S.V. (2007). d,l-Sulforaphane-induced cell death in human prostate cancer cells is regulated by inhibitor of apoptosis family proteins and Apaf-1. Carcinogenesis.

[101] Wiczk A., Hofman D., Konopa G., Herman-Antosiewicz A. (2012). Sulforaphane, a cruciferous vegetable-derived isothiocyanate, inhibits protein synthesis in human prostate cancer cells. Biochim. Biophys. Acta.

[102] Xiao D., Powolny A.A., Moura M.B., Kelley E.E., Bommareddy A., Kim S.H., Hahm E.R., Normolle D., van Houten B., Singh S.V. (2010). Phenethyl isothiocyanate inhibits oxidative phosphorylation to trigger reactive oxygen species-mediated death of human prostate cancer cells. J. Biol. Chem..

[103] Mi L., Gan N., Cheema A., Dakshanamurthy S., Wang X., Yang D.C., Chung F.L. (2009). Cancer preventive isothiocyanates induce selective degradation of cellular alpha- and beta-tubulins by proteasomes. J. Biol. Chem..

[104] Singh S.V., Srivastava S.K., Choi S., Lew K.L., Antosiewicz J., Xiao D., Zeng Y., Watkins S.C., Johnson C.S., Trump D.L. (2005). Sulforaphane-induced cell death in human prostate cancer cells is initiated by reactive oxygen species. J. Biol. Chem..

[105] Rodríguez-Berriguete G., Torrealba N., Ortega M.A., Martínez-Onsurbe P., Olmedilla G., Paniagua R., Guil-Cid M., Fraile B., Royuela M. (2015). Prognostic value of inhibitors of apoptosis proteins (IAPs) and caspases in prostate cancer: Caspase-3 forms and XIAP predict biochemical progression after radical prostatectomy. BMC Cancer.

[106] Lin H.P., Lin C.Y., Hsiao P.H., Wang H.D., Sheng Jiang S., Hsu J.M., Jim W.T., Chen M., Kung H.J., Chuu C.P. (2013). Difference in protein expression profile and chemotherapy drugs response of different progression stages of LNCaP sublines and other human prostate cancer cells. PLoS ONE.

[107] Raffo A.J., Perlman H., Chen M.W., Day M.L., Streitman J.S., Buttyan R. (1995). Overexpression of bcl-2 protects prostate cancer cells from apoptosis *in vitro* and confers resistance to androgen depletion *in vivo*. Cancer Res..

[108] Lin Y., Fukuchi J., Hiipakka R.A., Kokontis J.M., Xiang J. (2007). Up-regulation of Bcl-2 is required for the progression of prostate cancer cells from an androgen-dependent to an androgen-independent growth stage. Cell Res..

[109] Bauer J.J., Sesterhenn I.A., Mostofi F.K., McLeod D.G., Srivastava S., Moul J.W. (1996). Elevated levels of apoptosis regulator proteins p53 and bcl-2 are independent prognostic biomarkers in surgically treated clinically localized prostate cancer. J. Urol..

[110] Matsushima H., Kitamura T., Goto T., Hosaka Y., Homma Y., Kawabe K. (1997). Combined analysis with Bcl-2 and P53 immunostaining predicts poorer prognosis in prostatic carcinoma. J. Urol..

[111] Yong W.P., Innocenti F., Ratain M.J. (2006). The role of pharmacogenetics in cancer therapeutics. Br. J. Clin. Pharmacol..

[112] Gyrd-Hansen M., Meier P. (2010). IAPs: From caspase inhibitors to modulators of NF-kappaB, inflammation and cancer. Nat. Rev. Cancer.

[113] Berezovskaya O., Schimmer A.D., Glinskii A.B., Pinilla C., Hoffman R.M., Reed J.C., Glinsky G.V. (2005). Increased expression of apoptosis inhibitor protein XIAP contributes to anoikis resistance of circulating human prostate cancer metastasis precursor cells. Cancer Res..

[114] Seligson D.B., Hongo F., Huerta-Yepez S., Mizutani Y., Miki T., Yu H., Horvath S., Chia D., Goodglick L., Bonavida B. (2007). Expression of X-linked inhibitor of apoptosis protein is a strong predictor of human prostate cancer recurrence. Clin. Cancer Res..

[115] Zhu M.L., Kyprianou N. (2010). Role of androgens and the androgen receptor in epithelial-mesenchymal transition and invasion of prostate cancer cells. FASEB J..

[116] Nauseef J.T., Henry M.D. (2011). Epithelial-to-mesenchymal transition in prostate cancer: Paradigm or puzzle?. Nat. Rev. Urol..

[117] Shin D.Y., Lee W.S., Jung J.H., Hong S.H., Park C., Kim H.J., Kim G.Y., Hwang H.J., Kim G.S., Jung J.M. (2013). Flavonoids from Orostachys japonicus A. Berger inhibit the invasion of LnCaP prostate carcinoma cells by inactivating Akt and modulating tight junctions. Int. J. Mol. Sci..

[118] Tomlins S.A., Rhodes D.R., Perner S., Dhanasekaran S.M., Mehra R., Sun X.W., Varambally S., Cao X., Tchinda J., Kuefer R. (2005). Recurrent fusion of TMPRSS2 and ETS transcription factor genes in prostate cancer. Science.

[119] Gopalan A., Leversha M.A., Satagopan J.M., Zhou Q., Al-Ahmadie H.A., Fine S.W., Eastham J.A., Scardino P.T., Scher H.I., Tickoo S.K. (2009). TMPRSS2-ERG gene fusion is not associated with outcome in patients treated by prostatectomy. Cancer Res..

[120] Cai J., Kandagatla P., Singareddy R., Kropinski A., Sheng S., Cher M.L., Chinni S.R. (2010). Androgens induce functional CXCR4 through ERG factor expression in TMPRSS2-ERG fusion-positive prostate cancer cells. Transl. Oncol..

[121] Hsiao J.J., Ng B.H., Smits M.M., Wang J., Jasavala R.J., Martinez H.D., Lee J., Alston J.J., Misonou H., Trimmer J.S., Wright M.E. (2015). Androgen receptor and chemokine receptors 4 and 7 form a signaling axis to regulate CXCL12-dependent cellular motility. BMC Cancer.

[122] Sakao K., Vyas A.R., Chinni S.R., Amjad A.I., Parikh R., Singh S.V. (2015). CXCR4 is a novel target of cancer chemopreventative isothiocyanates in prostate cancer cells. Cancer Prev. Res..

[123] Labsch S., Liu L., Bauer N., Zhang Y., Aleksandrowicz E., Gladkich J., Schönsiegel F., Herr I. (2014). Sulforaphane and TRAIL induce a synergistic elimination of advanced prostate cancer stem-like cells. Int. J. Oncol..

[124] Peng X., Zhou Y., Tian H., Yang G., Li C., Geng Y., Wu S., Wu W. (2015). Sulforaphane inhibits invasion by phosphorylating ERK1/2 to regulate E-cadherin and CD44v6 in human prostate cancer DU145 cells. Oncol. Rep..

[125] Leong K.G., Gao W.Q. (2008). The Notch pathway in prostate development and cancer. Differentiation.

[126] Wang Z., Li Y., Banerjee S., Kong D., Ahmad A., Nogueira V., Hay N., Sarkar F.H. (2010). Down-regulation of Notch-1 and Jagged-1 inhibits prostate cancer cell growth, migration and invasion, and induces apoptosis via inactivation of Akt, mTOR, and NF-kappaB signaling pathways. J. Cell Biochem..

[127] Bin Hafeez B., Adhami V.M., Asim M., Siddiqui I.A., Bhat K.M., Zhong W., Saleem M., Din M., Setaluri V., Mukhtar H. (2009). Targeted knockdown of Notch1 inhibits invasion of human prostate cancer cells concomitant with inhibition of matrix metalloproteinase-9 and urokinase plasminogen activator. Clin. Cancer Res..

[128] Hahm E.R., Chandra-Kuntal K., Desai D., Amin S., Singh S.V. (2012). Notch activation is dispensable for d, l-sulforaphane-mediated inhibition of human prostate cancer cell migration. PLoS ONE.

[129] Kim S.H., Sehrawat A., Sakao K., Hahm E.R., Singh S.V. (2011). Notch activation by phenethyl isothiocyanate attenuates its inhibitory effect on prostate cancer cell migration. PLoS ONE.

[130] Sharpe B., Beresford M., Bowen R., Mitchard J., Chalmers A.D. (2013). Searching for prostate cancer stem cells: Markers and methods. Stem Cell Rev..

[131] Feitelson M.A., Arzumanyan A., Kulathinal R.J., Blain S.W., Holcombe R.F., Mahajna J., Marino M., Martinez-Chantar M.L., Nawroth R., Sanchez-Garcia I. (2015). Sustained proliferation in cancer: Mechanisms and novel therapeutic targets. Semin. Cancer Biol..

[132] Ojo D., Lin X., Wong N., Gu Y., Tang D. (2015). Prostate cancer stem-like cells contribute to the development of castration-resistant prostate cancer. Cancers.

[133] Vander Griend D.J., Litvinov I.V., Isaacs J.T. (2014). Conversion of androgen receptor signaling from a growth suppressor in normal prostate epithelial cells to an oncogene in prostate cancer cells involves a gain of function in c-Myc regulation. Int. J. Biol. Sci..

[134] Sankpal U.T., Goodison S., Abdelrahim M., Basha R. (2011). Targeting Sp1 transcription factors in prostate cancer therapy. Med. Chem..

[135] Zhang W., Meng Y., Liu N., Wen X.F., Yang T. (2015). Insights into chemoresistance of prostate cancer. Int. J. Biol. Sci..

[136] Zhao M., Jiang B., Gao F.H. (2011). Small molecule inhibitors of STAT3 for cancer therapy. Curr. Med. Chem..

[137] Bishop J.L., Thaper D., Zoubeidi A. (2014). The multifaceted roles of STAT3 signaling in the progression of prostate cancer. Cancers.

[138] Barton B.E., Karras J.G., Murphy T.F., Barton A., Huang H.F. (2004). Signal transducer and activator of transcription 3 (STAT3) activation in prostate cancer: Direct STAT3 inhibition induces apoptosis in prostate cancer lines. Mol. Cancer Ther..

[139] Yue P., Turkson J. (2009). Targeting STAT3 in cancer: How successful are we?. Expert Opin. Investig. Drugs.

[140] Pencik J., Schlederer M., Gruber W., Unger C., Walker S.M., Chalaris A., Marié I.J., Hassler M.R., Javaheri T., Aksoy O. (2015). STAT3 regulated ARF expression suppresses prostate cancer metastasis. Nat. Commun..

[141] Mora L.B., Buettner R., Seigne J., Diaz J., Ahmad N., Garcia R., Bowman T., Falcone R., Fairclough R., Cantor A. (2002). Constitutive activation of Stat3 in human prostate tumors and cell lines: Direct inhibition of Stat3 signaling induces apoptosis of prostate cancer cells. Cancer Res..

[142] Aaronson D.S., Muller M., Neves S.R., Chung W.C., Jayaram G., Iyengar R., Ram P.T. (2007). An androgen-IL-6-Stat3 autocrine loop re-routes EGF signal in prostate cancer cells. Mol. Cell Endocrinol..

[143] Siegmund S.A., Sommer J., Scheller J., Rose-John S., Garbers C. (2015). ID: 204: Interleukin-6-Induced and constitutive activation of signal transducer and activator of transcription 3 relies on protein kinase II activity. Cytokine.

[144] Hahm E.R., Singh S.V. (2010). Sulforaphane inhibits constitutive and interleukin-6-induced activation of signal transducer and activator of transcription 3 in prostate cancer cells. Cancer Prev. Res..

[145] Edlind M.P., Hsieh A.C. (2014). PI3K-AKT-mTOR signaling in prostate cancer progression and androgen deprivation therapy resistance. Asian J. Androl..

[146] Khor T.O., Keum Y.S., Lin W., Kim J.H., Hu R., Shen G., Xu C., Gopalakrishnan A., Reddy B., Zheng X. (2006). Combined inhibitory effects of curcumin and phenethyl isothiocyanate on the growth of human PC-3 prostate xenografts in immunodeficient mice. Cancer Res..

[147] Shankar S., Ganapathy S., Srivastava R.K. (2008). Sulforaphane enhances the therapeutic potential of TRAIL in prostate cancer orthotopic model through regulation of apoptosis, metastasis, and angiogenesis. Clin Cancer Res..

[148] Hudson T.S., Perkins S.N., Hursting S.D., Young H.A., Kim Y.S., Wang T.C., Wang T.T. (2012). Inhibition of androgen-responsive LNCaP prostate cancer cell tumor xenograft growth by dietary phenethyl isothiocyanate correlates with decreased angiogenesis and inhibition of cell attachment. Int. J. Oncol..

[149] Xiao D., Zeng Y., Choi S., Lew K.L., Nelson J.B., Singh S.V. (2005). Caspase-dependent apoptosis induction by phenethyl isothiocyanate, a cruciferous vegetable-derived cancer chemopreventive agent, is mediated by Bak and Bax. Clin Cancer Res..

[150] Xiao D., Lew K.L., Zeng Y., Xiao H., Marynowski S.W., Dhir R., Singh S.V. (2006). Phenethyl isothiocyanate-induced apoptosis in PC-3 human prostate cancer cells is mediated by reactive oxygen species-dependent disruption of the mitochondrial membrane potential. Carcinogenesis.

[151] Sakao K., Hahm E.R., Singh S.V. (2013). *In vitro* and *in vivo* effects of phenethyl isothiocyanate treatment on vimentin protein expression in cancer cells. Nutr Cancer..

[152] Singh S.V., Warin R., Xiao D., Powolny A.A., Stan S.D., Arlotti J.A., Zeng Y., Hahm E.R., Marynowski S.W., Bommareddy A. (2009). Sulforaphane inhibits prostate carcinogenesis and pulmonary metastasis in TRAMP mice in association with increased cytotoxicity of natural killer cells. Cancer Res..

[153] Chung F.L., Morse M.A., Eklind K.I., Lewis J. (1992). Quantitation of human uptake of the anticarcinogen phenethyl isothiocyanate after a watercress meal. Cancer Epidemiol Biomark. Prev..

[154] Xiao D., Singh S.V. (2010). Phenethyl isothiocyanate sensitizes androgen-independent human prostate cancer cells to docetaxel-induced apoptosis *in vitro* and *in vivo*. Pharm. Res..

[155] Barve A., Khor T.O., Hao X., Keum Y.S., Yang C.S., Reddy B., Kong A.N. (2008). Murine prostate cancer inhibition by dietary phytochemicals—Curcumin and phenyethylisothiocyanate. Pharm. Res..

[156] Myzak M.C., Tong P., Dashwood W.M., Dashwood R.H., Ho E. (2007). Sulforaphane retards the growth of human PC-3 xenografts and inhibits HDAC activity in human subjects. Exp. Biol. Med. (Maywood).

[157] Bommareddy A., Hahm E.R., Xiao D., Powolny A.A., Fisher A.L., Jiang Y., Singh S.V. (2009). Atg5 regulates phenethyl isothiocyanate-induced autophagic and apoptotic cell death in human prostate cancer cells. Cancer Res..

[158] Traka M.H., Spinks C.A., Doleman J.F., Melchini A., Ball R.Y., Mills R.D., Mithen R. (2010). F The dietary isothiocyanate sulforaphane modulates gene expression and alternative gene splicing in a PTEN null preclinical murine model of prostate cancer. Mol. Cancer..

[159] Xiao D., Singh S.V. (2010). p66Shc is indispensable for phenethyl isothiocyanate-induced apoptosis in human prostate cancer cells. Cancer Res..

[160] Li R.W., Li C., Wang T.T. (2013). Transcriptomic alterations in human prostate cancer cell LNCaP tumor xenograft modulated by dietary phenethyl isothiocyanate. Mol. Carcinog..

[161] Cho H.J., Lim D.Y., Kwon G.T., Kim J.H., Huang Z., Song H., Oh Y.S., Kang Y.H., Lee K.W., Dong Z. (2016). Benzyl isothiocyanate inhibits prostate cancer development in the transgenic adenocarcinoma mouse prostate (TRAMP) model, which is associated with the induction of cell cycle G1 arrest. Int. J. Mol. Sci..

[162] Srivastava S.K., Xiao D., Lew K.L., Hershberger P., Kokkinakis D.M., Johnson C.S., Trump D.L., Singh S.V. (2003). Allyl isothiocyanate, a constituent of cruciferous vegetables, inhibits growth of PC-3 human prostate cancer xenografts *in vivo*. Carcinogenesis.

[163] Halvorsen O.J. (2008). Molecular and prognostic markers in prostate cancer. A study of cell-cycle regulators, angiogenesis and candidate markers. APMIS Suppl..

[164] Liang J., Slingerland J.M. (2003). Multiple roles of the PI3K/PKB (Akt) pathway in cell cycle progression. Cell Cycle.

[165] Srinivasula S.M., Ashwell J.D. (2008). IAPs: What’s in a name?. Mol. Cell..

[166] Deshmukh D., Qiu Y. (2015). Role of PARP-1 in prostate cancer. Am. J. Clin. Exp. Urol..

[167] Choi K.S. (2012). Autophagy and cancer. Exp. Mol. Med..

[168] Nandana S., Chung L.W. (2014). Prostate cancer progression and metastasis: Potential regulatory pathways for therapeutic targeting. Am. J. Clin. Exp. Urol..

[169] Ganguly S.S., Li X., Miranti C.K. (2014). The host microenvironment influences prostate cancer invasion, systemic spread, bone colonization, and osteoblastic metastasis. Front Oncol..

[170] Brooks S.A., Lomax-Browne H.J., Carter T.M., Kinch C.E., Hall D.M. (2010). Molecular interactions in cancer cell metastasis. Acta Histochem..

[171] Tenta R., Katopodis H., Chatziioannou A., Pilalis E., Calvo E., Luu-The V., Labrie F., Kolisis F., Koutsilieris M. (2007). Microarray analysis of survival pathways in human PC-3 prostate cancer cells. Cancer Genom. Proteom..

[172] Abbott A. (2006). Cancer: The root of the problem. Nature.

[173] Bono A.V., Celato N., Cova V., Salvadore M., Chinetti S., Novario R. (2002). Microvessel density in prostate carcinoma. Prostate Cancer Prostatic. Dis..

[174] Mukherji D., Temraz S., Wehbe D., Shamseddine A. (2013). Angiogenesis and anti-angiogenic therapy in prostate cancer. Crit. Rev. Oncol. Hematol..

[175] Mucci L.A., Powolny A., Giovannucci E., Liao Z., Kenfield S.A., Shen R., Stampfer M.J., Clinton S.K. (2009). Prospective study of prostate tumor angiogenesis and cancer-specific mortality in the health professionals follow-up study. J. Clin. Oncol..

